# Synthesis and Antibacterial Activity of Benzo[4,5]isothiazolo[2,3-*a*]pyrazine-6,6-dioxide Derivatives

**DOI:** 10.3390/molecules22111889

**Published:** 2017-11-04

**Authors:** Jatinder P. Bassin, Michelle J. Botha, Rajesh Garikipati, Madhu Goyal, Lee Martin, Amit Shah

**Affiliations:** 1 School of Life and Medical Sciences, University of Hertfordshire, Hatfield AL10 9AB, UK; m.botha@herts.ac.uk (M.J.B.); r.garikipati4@gmail.com (R.G.); amit_pharmacy@hotmail.co.uk (A.S.); 2 School of Science and Technology, Nottingham Trent University, Clifton Lane, Clifton, Nottingham NG11 8NS, UK; lee.martin@ntu.ac.uk

**Keywords:** chalcones, benzo[4,5]isothiazolo[2,3-*a*]pyrazine-6,6-dioxides, *Bacillus subtilis*, *Escherichia coli*, *Proteus vulgaris*, *Staphylococcus aureus*

## Abstract

Using a routine procedure, a number of derivatives of the benzo[4,5]isothiazolo[2,3-*a*]pyrazine-6,6-dioxide ring system have been synthesized from readily available starting materials. A series of chalcones were synthesized, which were subsequently reacted with chlorosulfonic acid to generate chalcone sulfonyl chlorides. The chalcone sulfonyl chlorides were then treated with bromine to generate dibromo chalcone sulfonyl chlorides. These were subsequently reacted with 1,2-diaminopropane and 2-methyl-1,2-diaminopropane in boiling ethanol resulting in compounds **2**–**10** and **11**–**19** respectively, in 12–80% yields. The products were characterized by spectral analysis and the definitive structure of compound **11** was determined by X-ray crystallography. The synthesized compounds were screened for potential antibacterial properties against *Bacillus subtilis*, *Escherichia coli*, *Proteus vulgaris* and *Staphylococcus aureus*.

## 1. Introduction

Antibacterial resistance has provoked an urgent need for novel antimicrobial agents. There has been a significant decline in the number of new antibacterial agents being released onto the market [1]. Research by the Infectious Disease Society of America has stated that there needs to be a minimum of ten new antimicrobial agents developed within the next eight years [2]. According to Theueretzbacher, the development of antibacterial agents has slowed considerably, and the main research in developing this group of drugs is being carried out in the pharma industry and academic institutions [3,4]. A possible reason for this could be that there are few targets available for selective toxic agents to act on. The expected success of high-throughput screening and combinatorial libraries did not live up to expectations [5]. The development of antimicrobial resistance has generated a huge demand for the successful production of novel pharmaceutically active compounds.

**Figure 1:**
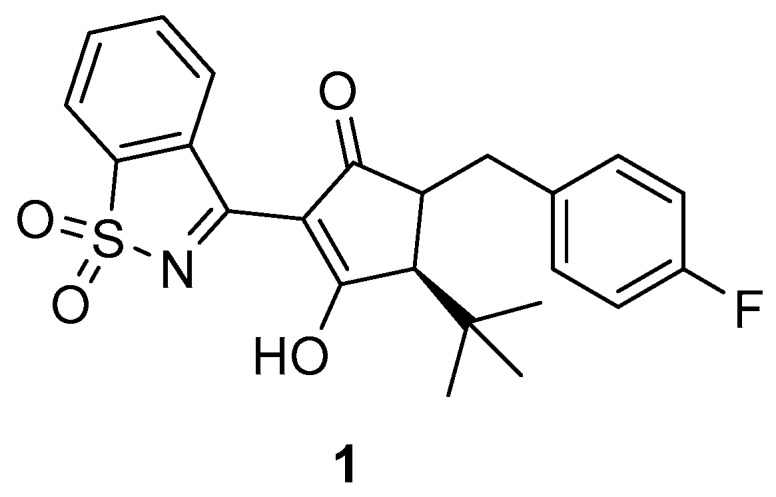
Structure of a benzo[*d*]isothiazole-1,1-dioxide **1**.

Due to the emergence of superbugs, such as methicillin resistant S*taphylococcus aureus* (MRSA), many antibiotics are no longer effective therapeutic agents. Although MRSA and vancomycin resistance appears to be stabilizing, there are new problems occurring [6]. Heterocycles are an enormously diverse group of compounds that are widely distributed in nature, and are found in many pharmaceuticals. Heterocycles are easily manipulated in organic synthetic routes; the compounds can be easily modified to increase or decrease reactivity [7]. Heterocycles are used extensively as intermediates in reactions and as building blocks in synthesis. Benzoisothiazole **1** rings (see Figure 1) are found in many chemically interesting compounds and derivatives of **1** have been synthesized. Derivatives of **1** have shown varied biological activity [8].

Benzo[*d*]isothiazole combined with an arylidene moiety (a methylene derivative of an aryl group) with a different substituent, such as a methyl group and a fluorine atom, have displayed cytotoxicity against human CD4^+^ lymphocytes. These compounds inhibited the growth of leukemia cell lines and also showed anti-proliferative activity against solid tumor-derived cell lines [8]. Vicini and colleagues investigated benzoisothiazole hydrazone derivatives as possible antiviral agents. The investigation focused on the structural requirements that are essential for anti-HIV activity and how modifying the alkene chain length between the benzoisothiazole ring and the hydrazone group would increase or decrease any possible anti-HIV activity. Although many of the compounds did show promising results, the mechanism of action remains to be elucidated [9]. Benzothiazole derivatives have also been found to inhibit human cyclooxygenase-2-enzymes (COX-2) [10].

Recent research in CNS-mediated diseases has given rise to discovery of a family of serotonin (5-HT) receptors, 5-HT_6_. In vivo studies have shown that inhibition of 5-HT_6_ has a significant positive impact on cognitive impairment. Sufferers from diseases such as Alzheimer’s and schizophrenia could potentially have an effective treatment within the next decade [11,12,13]. In 2012, researchers synthesized a group of benzoisothiazole derivatives that contained an *N*,*N*-dimethylformamide group, of which two of the compounds displayed promising activity as potential 5-HT_6_ antagonists [14]. Chikalia and colleagues synthesized a library of twenty benzimidazole-1,3,4-oxadiazole derivatives which showed anti-tuberculosis activity at concentrations as low as 4 μg [15]. Pyrazine derivatives have been extensively reviewed in the literature [16] and have displayed a wide range of antimicrobial properties [17]. Pyrazine derivatives also play an important role in the food industry [18]. Recent research on derivatives of bisbenzothiazolyl-pyridines and -pyrazine displayed strong antiproliferative activity which has been suggested to work by exerting oxidative stress on the cancer cells [17].

## 2. Results and Discussion

The biological properties of heterocycles, which include nitrogen, oxygen and sulfur, have been reported extensively over the last fifty years [19]. The literature search revealed that only a small number of patents report synthetic routes to the isothiazolo[2,3-a]pyrazine ring system [20,21]. The synthesis started with by reacting 3,4-dimethoxybenzaldehyde with substituted acetophenones following the known literature method to generate a number of chalcones [22]. The chalcones were initially reacted with chlorosulfonic acid to yield the sulfonyl chlorides which were subsequently reacted with bromine in glacial acetic acid resulting in the dibromochalcone sulfonyl chlorides. These dibromochalcones were reacted with 1,2-diaminopropane and 2-methyl-1,2-diaminopropane to afford benzo[4,5]isothiazolo[2,3-*a*]pyrazine derivatives **2**–**10** and **11**–**19** respectively.

**Scheme 1:**
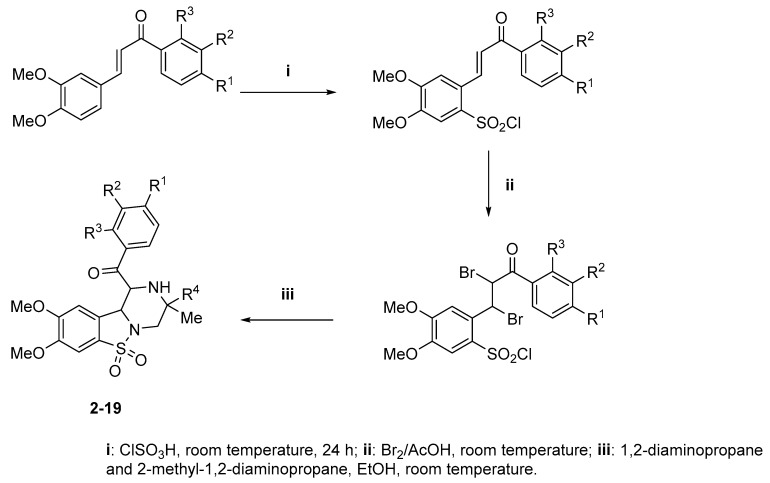
Synthesis of benzo[4,5]isothiazolo[2,3-*a*]pyrazine-6,6-dioxide derivatives.

The isolated products were identified on the basis of their spectral (IR, NMR and MS) data as the respective benzo[4,5]isothiazolo[2,3-*a*]pyrazine-6,6-dioxide derivatives **2**–**19** (Scheme 1 and Table 1). For example, the ^1^H-NMR spectrum of compound **12** showed a doublet at δ 7.86 and 7.19 with a coupling constant of 8.3 Hz, which is consistent with para substitution in the benzoyl ring. Two singlets were observed at δ 7.19 and δ 6.36 for the aromatic protons in the benzoisothiazole ring. The two methoxy methyl protons appeared as singlets at δ 3.89 and δ 3.45 and the two methyl protons of the pyrazine ring appeared as singlets at δ 1.39 and δ 1.22. The methine protons appeared at δ 3.64 and δ 2.94 as doublets with a coupling constant of 12.8 Hz, consistent with axial vicinal protons [23], and the methylene hydrogens showed up at δ 4.55 and δ 4.47 as doublets with a coupling constant of 9.2 Hz. 

**Figure 2:**
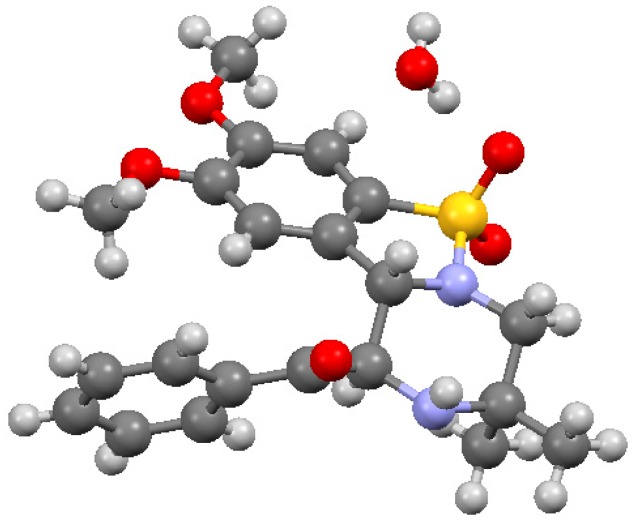
X-Ray crystal structure of compound **11** (dark grey = carbon; light grey = hydrogen; red = oxygen; blue = nitrogen; yellow = sulfur).

The definitive structure of compound **11** was confirmed by X-ray analyses (see Figure 2). The X-ray structure clearly confirmed that the two methyl groups were on the carbon atom next to the NH group in the pyrazine ring. All the compounds generated were reacted under the same reaction conditions. Using NMR data combined with the X-ray data, the structures for all eighteen compounds were correctly elucidated.

Out of all the compounds, compound **7** was the only compound that inhibited the growth of all the four bacteria tested. The growth of *Escherichia coli*, *Proteus vulgaris* and *Staphylococcus aureus* was inhibited at a concentration of 1.6 mg·mL^−1^ or above (Table 2). As the concentration of compound **7** decreased from 1.6 mg·mL^−1^ down to the lowest concentration of 0.052 mg·mL^−1^ the inhibitory effect of the compound decreased rapidly as seen by the increase in absorbance measured at 590 nm and the MTT (3-(4,5-dimethylthiazol-2-yl)-2,5-diphenyltetrazolium bromide) assay. This compound also showed activity against *Bacillus subtilis*, though the growth was inhibited at a lower concentration (0.833 mg/mL) as shown by decrease in absorbance measured at 590 nm. Compound **7** had one methyl group present on the fused pyrazine ring and a chlorine atom in the *meta* position on the benzene ring. There was no visible trend or similarity in results that correlated to the number of methyl groups on the pyrazine ring. There was no visible correlation between activity and halogen present; however there is much evidence in the literature to support the potency enhancing effects of halogen substituents [15]. The addition of halogens are frequently employed to increase antimicrobial activity, however with the exception of compound **7**, there was no significant difference in activity when varying the substituent group. 

## 3. Experimental Section

### 3.1. General Information

All chemicals were purchased from Sigma Aldrich (St. Louis, MO, USA) and were used without any further purification. Melting points were determined using a Gallenkamp melting point apparatus (Thermo Fisher Scientific, Paisley, UK) and are uncorrected. The NMR spectra were recorded using a 600 MHz spectrometer (JEOL Co. Ltd., Tokyo, Japan) with tetramethylsilane as the internal standard and solvents as indicated. Chemical shifts were measured in ppm (δ) relative to TMS (0.00 ppm). Coupling constants (*J*) are reported in Hertz (Hz). The following abbreviations are used to describe the signal multiplicities: s (singlet), d (doublet), t (triplet), q (quartet) and m (multiplet). LC-MS spectra were obtained with a spectrometer equipped with an Electron Spray Ionisation (ESI) source (Varian: 210 LC pumps × 2, 1200 L Quadrapole MS/MS, 410 autosampler) (Varian (now Agilent), Oxford, UK) using a gradient solvent system of A: Water/0.1% formic acid and B: acetonitrile/0.1% formic acid. Infrared spectra were recorded with a Varian 800 FT-IR spectrophotometer (Varian) as KBr discs. 

### 3.2. Synthesis of Chalcones

The chalcones were synthesized by the well-established procedure using acetophenones and 3,4-dimethoxybenzaldehyde [22,24,25,26,27,28].

### 3.3. General Procedure for Synthesis of Chalcone Sulfonyl Chlorides

The chalcones (10 g; 0.032 mol) were added in portions to stirred chlorosulfonic acid (37.67 g; 0.32 mol) in an ice bath. After the addition was complete, the reaction mixture was left stirring at room temperature. Progress of the reaction was monitored by thin layer chromatography (TLC) (Fisher Scientific, Loughborough, UK When the reaction was complete (24 h), the mixture was poured slowly over ice to remove excess chlorosulfonic acid. The sulfonyl chlorides were filtered by suction filtration and washed with a cold water acetonitrile mixture. The resulting precipitate was considered pure enough to be used in subsequent reactions by TLC analysis.

### 3.4. General Procedure for the Synthesis of Dibromo Chalcone Sulfonyl Chlorides

The crude chalcone sulfonyl chloride (10 g; 0.032 mol) was added to glacial acetic acid (125 mL) with stirring. The resulting mixture was stirred at room temperature and to the stirred mixture was added bromine (20.48 g; 0.13 mol) dissolved in 50 mL glacial acetic acid. The mixture was stirred until a precipitate was formed, which was filtered and washed with cold glacial acetic acid.

### 3.5. General Method for Synthesis of Benzo[4,5]isothiazolo[2,3-a]pyrazin-1-yl)(phenyl) Methanones (***2***–***10***)

To a stirred solution of the chalcone dibromo sulphonyl chloride (1 g) in ethanol (25 mL), was added 1,2-diaminopropane (2 mole equivalent). The reaction mixture was warmed in a water bath for 15 min until all the solids had dissolved. The reaction mixture was allowed to cool to room temperature and the resulting solid was filtered and air dried. The product was purified by recrystallization from ethanol.

*(8,9-Dimethoxy-3-methyl-6,6-dioxido-2,3,4,10b-tetrahydro-1H-benzo[4,5]isothiazolo[2,3-a]pyrazin-1-yl)(Phenyl)methanone* (**2**). Yellow crystals; yield 29%; m.p. 219–220 °C. IR: (KBr ν cm^−1^) 3337 (NH); 1681 (C=O), 1287, 1176 (SO_2_). ^1^H-NMR (600 MHz, CDCl_3_) δ ppm 1.15 (d, *J* = 6.42 Hz, 3 H), 2.79–2.88 (m, 1 H), 3.14–3.36 (m, 1 H), 3.48 (s, 3 H), 3.89 (s, 3 H), 3.90–3.94 (m, 1 H), 4.42 (d, *J* = 10.09 Hz, 1 H), 4.64 (d, *J* = 10.09 Hz, 1 H), 6.40 (s, 1 H), 7.21 (s, 1 H), 7.43 (t, *J* = 7.79 Hz, 2 H), 7.58 (t, *J* = 7.34 Hz, 1 H), 7.90 (d, *J* = 8.25 Hz, 2 H). ^13^C-NMR (CDCl_3_) δ ppm 18.90, 47.30, 48.29, 56.02, 56.40, 58.49, 62.49, 102.81, 107.34, 126.42, 127.87, 129.07, 129.17, 134.51, 135.54, 135.51, 150.47, 152.62, 198.30. MS (ESI *m*/*z*) 402.3 [M]^+^. Anal. Calcd. For: C_20_H_22_N_2_O_5_S: C, 59.69; H, 5.51. Found: C, 59.35; H, 5.60.

*(8,9-Dimethoxy-3-methyl-6,6-dioxido-2,3,4,10b-tetrahydro-1H-benzo[4,5]isothiazolo[2,3-a]pyrazin-1-yl)(p-tolyl)methanone* (**3**). White crystals; yield 47%; m.p. 180–181 °C. IR: (KBr ν cm^−1^) 3447 (NH), 1663 (C=O), 1284, 1140 (SO_2_). ^1^H-NMR (600 MHz, CDCl_3_) δ ppm 1.53 (d, *J* = 6.19 Hz, 3 H), 2.42 (m, 3 H), 3.13 (m, 1 H), 3.50 (d, *J* = 14.43 Hz, 1 H), 3.77 (dd, *J* = 14.43, 1.37 Hz, 1 H), 3.94 (s, 3 H), 3.98 (s, 3 H), 4.29 (d, *J* = 11.00 Hz, 1 H), 6.80 (s, 1 H), 7.26 (m, 3 H), 7.68 (d, *J* = 8.25 Hz, 2 H). ^13^C-NMR (151 MHz, CDCl_3_) δ ppm 22.48, 38.76, 45.67, 55.47, 56.54, 56.67, 56.95, 102.97, 105.06, 123.14, 125.38, 126.06, 129.77, 130.15, 130.23, 133.17, 142.62, 150.81, 153.59, 166.65. MS (ESI *m*/*z*) 416.3 [M]^+^. Anal. Calcd. For: C_21_H_24_N_2_O_5_S: C, 60.56; H, 5.81. Found: C, 60.60; H, 5.72.

*(8,9-Dimethoxy-3-methyl-6,6-dioxido-2,3,4,10b-tetrahydro-1H-benzo[4,5]isothiazolo[2,3-a]pyrazin-1-yl)(4-fluorophenyl)methanone* (**4**). Yellow crystals; yield 30%; m.p. 181–182 °C. IR: (KBr ν cm^−1^) 3297 (NH), 1677 (C=O), 1290, 1144 (SO_2_). ^1^H-NMR (151 MHz, CDCl_3_) δ ppm 1.14 (d, *J* = 6.42 Hz, 3 H), 3.55 (s, 3 H), 3.89 (s, 3 H), 3.92 (dd, *J* = 14.67, 3.67 Hz, 1 H), 4.34 (d, *J* = 10.09 Hz, 1 H), 4.65 (d, *J* = 9.17 Hz, 1 H), 6.45 (s, 1 H), 7.10 (t, *J* = 8.71 Hz, 2 H), 7.21 (s, 1 H), 7.95 (m, 2 H). ^13^C-NMR (151 MHz, CDCl_3_) δ ppm 18.93, 47.40, 48.28, 56.25, 58.23, 59.07, 62.61, 102.86, 107.26, 116.21, 116.36, 126.47, 127.92, 131.92, 131.91, 150.54, 152.70, 167.37, 196.74. MS (ESI *m*/*z*) 420.2 [M]^+^. Anal. Calcd. For: C_20_H_21_FN_2_O_5_S: C, 57.13; H, 5.03. Found: C, 57.23; H, 4.95.

*(8,9-Dimethoxy-3-methyl-6,6-dioxido-2,3,4,10b-tetrahydro-1H-benzo[4,5]isothiazolo[2,3-a]pyrazin-1-yl)(4-chlorophenyl)methanone* (**5**). Yellow crystals; yield 43%; m.p. 204–205 °C. IR: (KBr ν cm^−1^) 3468 (NH), 1672 (C=O), 1284, 1142 (SO_2_); ^1^H-NMR (600 MHz, CDCl_3_) δ ppm 1.52 (d, *J* = 6.87 Hz, 3 H), 2.99 (dd, *J* = 14.43, 9.62 Hz, 1 H), 3.14 (dd, *J* = 14.78, 11.34 Hz, 1 H), 3.45 (d, *J* = 15.12 Hz, 1 H), 3.77 (dd, *J* = 14.43, 1.37 Hz, 1 H), 3.95 (m, 3 H), 3.98 (m, 3 H), 4.28 (d, *J* = 11.68 Hz, 1 H), 6.79 (s, 1 H), 7.25 (s, 1 H), 7.42 (m, 2 H), 7.73 (d, *J* = 8.25 Hz, 2 H). ^13^C-NMR (151 MHz, CDCl_3_) δ ppm 22.58, 38.68, 45.70, 55.54, 56.55, 56.66, 56.70, 102.93, 104.95, 115.69, 115.83, 126.04, 128.98, 129.03, 129.89, 136.72, 150.74, 153.53, 166.84. MS (ESI *m*/*z*) 436.2 [M]^+^. Anal. Calcd. For: C_20_H_21_ClN_2_O_5_S: C, 54.98; H, 4.84. Found: C, 55.13; H, 4.75.

*(8,9-Dimethoxy-3-methyl-6,6-dioxido-2,3,4,10b-tetrahydro-1H-benzo[4,5]isothiazolo[2,3-a]pyrazin-1-yl)(4-bromophenyl)methanone* (**6**). Yellow crystals; yield 48%; m.p. 204–205 °C. IR (KBr ν cm^−1^): 3458 (NH), 1672 (C=O), 1284, 1141 (SO_2_). ^1^H-NMR (600 MHz, CDCl_3_) δ ppm 1.52 (d, *J* = 6.87 Hz, 3 H), 2.99 (dd, *J* = 14.43, 9.62 Hz, 1 H), 3.14 (dd, *J* = 14.78, 11.34 Hz, 1 H), 3.44 (d, *J* = 15.12 Hz, 1 H), 3.77 (d, *J* = 15.12 Hz, 1 H), 3.98 (m, 3 H), 3.98 (s, 3 H), 4.28 (d, *J* = 11.00 Hz, 1 H), 6.78 (s, 1 H), 7.25 (s, 1 H), 7.58 (d, *J* = 8.94 Hz, 2 H), 7.66 (m, 2 H). ^13^C-NMR (151 MHz, CDCl_3_) δ ppm 21.44, 22.63, 38.71, 45.73, 55.65, 56.28, 56.63, 102.89, 105.01, 126.01, 126.88, 129.47, 130.12, 137.88, 140.57, 150.66, 153.49, 167.93. MS (ESI *m*/*z*) 480.4 [M]^+^. Anal. Calcd. For: C_20_H_21_BrN_2_O_5_S: C, 49.90; H, 4.40. Found: C, 49.85; H, 4.36.

*(8,9-Dimethoxy-3-methyl-6,6-dioxido-2,3,4,10b-tetrahydro-1H-benzo[4,5]isothiazolo[2,3-a]pyrazin-1-yl)(3-chlorophenyl)methanone* (**7**). Light yellow crystals; yield 79%; m.p. 194–195 °C. IR (KBr ν cm^−1^): 1691 (C=O), 1270, 1158 (SO_2_). ^1^H-NMR (600 MHz, CDCl_3_) δ ppm 1.53 (d, *J* = 6.87 Hz, 3 H), 3.00 (dd, *J* = 14.43, 9.62 Hz, 1 H), 3.44 (m, 1 H), 3.78 (dd, *J* = 14.43, 1.37 Hz, 1 H), 3.95 (s, 3 H), 3.99 (m, 3 H), 4.29 (d, *J* = 11.00 Hz, 1 H), 6.80 (s, 1 H), 7.25 (s, 1 H), 7.39 (m, 1 H), 7.43 (m, 1 H), 7.63 (m, 1 H), 7.78 (t, *J* = 1.72 Hz, 1 H). ^13^C-NMR (151 MHz, CDCl_3_) δ ppm 14.87, 41.86, 46.31, 56.54, 56.65, 56.78, 56.97, 67.41, 110.98, 111.87, 112.01, 112.30, 127.15, 129.00, 130.45, 131.31, 134.17, 134.27, 134.94, 135.44, 135.79, 149.16, 149.30, 154.75, 188.68. MS (ESI *m*/*z*) 436.2 [M]^+^. Anal. Calcd. For: C_20_H_21_ClN_2_O_5_S: C, 54.98; H, 4.84. Found: C, 55.23; H, 4.92.

*(8,9-Dimethoxy-3-methyl-6,6-dioxido-2,3,4,10b-tetrahydro-1H-benzo[4,5]isothiazolo[2,3-a]pyrazin-1-yl)(3-bromophenyl)methanone* (**8**). Yellow crystals; yield 50%; m.p. 189–190 °C. IR (KBr ν cm^−1^): 3455 (NH), 1686 (C=O), 1290, 1140 (SO_2_). ^1^H-NMR (600 MHz, CDCl_3_) δ ppm 1.15 (d, *J* = 6.42 Hz, 3 H), 2.84 (m, 1 H), 3.27 (m, 1 H), 3.48 (s, 3 H), 3.89 (m, 3 H), 4.42 (d, *J* = 10.09 Hz, 1 H), 4.64 (d, *J* = 10.09 Hz, 1 H), 6.40 (s, 1 H), 7.21 (m, 1 H), 7.43 (t, *J* = 7.79 Hz, 2 H), 7.58 (t, *J* = 7.34 Hz, 2 H). ^13^C-NMR (151 MHz, CDCl_3_) δ ppm 18.71, 47.37, 48.36, 58.32, 58.21, 58.54, 62.85, 102.91, 107.23, 123.45, 126.45, 127.78, 127.72, 127.82, 131.85, 132.20, 137.22, 150.59, 152.77, 197.14. MS (ESI *m*/*z*) 482.7 [M]^+^. Anal. Calcd. For: C_20_H_21_BrN_2_O_5_S: C, 49.90; H, 4.40. Found: C, 49.80; H, 4.45.

*(8,9-Dimethoxy-3-methyl-6,6-dioxido-2,3,4,10b-tetrahydro-1H-benzo[4,5]isothiazolo[2,3-a]pyrazin-1-yl)(2-chlorophenyl)methanone* (**9**). Yellow crystals; yield 46%; m.p. 215–216 °C. IR (KBr ν cm^−1^): 3388 (NH), 1683 (C=O), 1290, 1190 (SO_2_). ^1^H-NMR (600 MHz, CDCl_3_) δ ppm 1.54 (d, *J* = 6.87 Hz, 3 H), 3.09 (dd, *J* = 14.43, 8.94 Hz, 1 H), 3.15 (dd, *J* = 14.78, 11.34 Hz, 1 H), 3.31 (d, *J* = 15.12 Hz, 1 H), 3.79 (d, *J* = 14.43 Hz, 1 H), 3.92 (s, 3 H), 3.94 (s, 3 H), 4.07 (m, 1 H), 4.66 (d, *J* = 11.00 Hz, 1 H), 7.24 (s, 1 H), 7.39 (d, *J* = 4.81 Hz, 2 H), 7.62 (d, *J* = 8.25 Hz, 2 H). ^13^C-NMR (151 MHz, CDCl_3_) δ ppm 22.54, 38.59, 45.68, 55.52, 56.55, 56.66, 56.83, 76.87, 77.08, 77.29, 102.97, 104.99, 126.09, 128.30, 128.96, 129.84, 136.51, 138.94, 150.79, 153.57, 166.84, 182.68, 182.36. MS (ESI *m*/*z*) 436.5 [M]^+^. Anal. Calcd. For: C_20_H_21_ClN_2_O_5_S: C, 54.98; H, 4.84. Found: C, 55.10; H, 5.02.

*(8,9-Dimethoxy-3-methyl-6,6-dioxido-2,3,4,10b-tetrahydro-1H-benzo[4,5]isothiazolo[2,3-a]pyrazin-1-yl)(2-bromophenyl)methanone* (**10**). Yellow crystals; yield 75%; m.p. 195–196 °C. IR (KBr ν cm^−1^): 3084 (NH), 1682 (C=O), 1286, 1123 (SO_2_). ^1^H-NMR (600 MHz, CDCl_3_) δ ppm 1.15 (d, *J* = 6.42 Hz, 3 H), 3.59 (s, 3 H), 3.92 (dd, *J* = 14.67, 3.67 Hz, 1 H), 3.89 (s, 3 H), 3.92 (m, 1 H), 4.32 (d, *J* = 9.17 Hz, 1 H), 4.66 (d, *J* = 10.09 Hz, 1 H), 6.44 (s, 1 H), 7.21 (s, 1 H), 7.29 (t, *J* = 7.79 Hz, 1 H), 7.69 (d, *J* = 8.25 Hz, 1 H), 7.78 (d, *J* = 7.34 Hz, 1 H), 8.07 (s, 1 H). ^13^C-NMR (151 MHz, CDCl_3_) δ ppm 18.83, 47.27, 48.42, 56.15, 58.07, 62.80 (s), 102.92, 107.25, 123.45, 126.45, 127.75, 130.58, 131.85, 137.24, 150.61, 152.76, 197.03. MS (ESI *m*/*z*) 480.4 [M]^+^. Anal. Calcd. For: C_20_H_21_BrN_2_O_5_S: C, 49.90; H, 4.40. Found: C, 50.10; H, 4.51.

### 3.6. General Method for Synthesis of Benzo[4,5]isothiazolo[2,3-a]pyrazin-1-yl)(phenyl) Methanones (***11***–***19***)

To a stirred solution of chalcone dibromo sulphonyl chloride (1 g) in ethanol (25 mL), was added 2-methyl-1,2-diaminopropane (2 mole equivalent). The reaction mixture was warmed in a water bath for 10–15 min until all the solids had dissolved. The reaction mixture was allowed to cool to room temperature and the resulting solid was filtered and air dried. The product was purified by recrystallization from ethanol. 

*(8,9-Dimethoxy-3,3-dimethyl-6,6-dioxido-2,3,4,10b-tetrahydro-1H-benzo[4,5]isothiazolo [2,3-a]pyrazin-1-yl)(phenyl)methanone* (**11**). White needle shaped crystals; yield 34%; m.p. 163–164 °C. IR (KBr ν cm^−1^): 3420 (NH), 1676 (C=O), 1277, 1141 (SO_2_). ^1^H-NMR (600 MHz, CDCl_3_) δ ppm 1.24 (s, 3 H), 1.41 (s, 3 H), 2.96 (d, *J* = 12.84 Hz, 1 H), 3.46 (s, 3 H), 3.89 (s, 3 H), 3.66 (d, *J* = 12.84 Hz, 1 H), 3.78 (m, 1 H), 4.51 (m, 1 H), 4.59 (m, 1 H), 6.38 (s, 1 H), 7.22 (s, 1 H), 7.48 (m, 2 H), 7.61 (m, 1 H), 7.98 (d, *J* = 7.34 Hz, 2 H). ^13^C-NMR (151 MHz, CDCl_3_) δ ppm 16.98, 18.39, 33.43, 56.43, 56.59, 58.72, 71.70, 102.94, 104.95, 124.29, 125.66, 126.34, 127.99, 128.80, 129.20, 130.85, 131.49, 131.89, 131.97, 132.10, 135.72, 147.20, 153.35, 165.30, 192.55. MS (ESI *m*/*z*) 416.3 [M]^+^. Anal. Calcd. For: C_21_H_24_N_2_O_5_S: C, 60.56; H, 5.81. Found: C, 60.60; H, 5.82.

*(8,9-Dimethoxy-3,3-dimethyl-6,6-dioxido-2,3,4,10b-tetrahydro-1H-benzo[4,5]isothiazolo[2,3-a]pyrazin-1-yl)(p-tolyl)methanone* (**12**). Light yellow crystals; yield 64%; m.p. 205–206 °C. IR (KBr ν cm^−1^): 3312 (NH), 1660 (C=O), 1275, 1177 (SO_2_). ^1^H-NMR (600 MHz, CDCl_3_) δ ppm 1.22 (s, 3 H), 1.39 (s, 3 H), 2.40 (s, 3 H), 2.94 (d, *J* = 12.84 Hz, 1 H), 3.45 (s, 3 H), 3.64 (d, *J* = 12.84 Hz, 1 H), 3.89 (s, 3 H), 4.47 (d, *J* = 9.17 Hz, 1 H), 4.55 (m, 1 H), 6.36 (s, 1 H), 7.21 (s, 1 H), 7.25 (d, *J* = 8.25 Hz, 2 H), 7.86 (d, *J* = 8.25 Hz, 2 H). ^13^C-NMR (151 MHz, CDCl_3_) δ ppm 20.23, 23.07, 28.32, 49.66, 49.95, 56.08, 56.44, 58.34, 58.52, 102.89, 106.71, 123.49, 126.89, 127.73, 130.64, 132.03, 137.24, 137.33, 150.66, 152.85, 197.35. MS (ESI *m*/*z*) 430.3 [M]^+^. Anal. Calcd. For: C_22_H_26_N_2_O_5_S: C, 61.38; H, 6.09. Found: C, 61.30; H, 6.10. 

*(8,9-Dimethoxy-3,3-dimethyl-6,6-dioxido-2,3,4,10b-tetrahydro-1H-benzo[4,5]isothiazolo[2,3-a]pyrazin-1-yl)(4-fluorophenyl)methanone* (**13**). White crystals; yield 12%; m.p. 175–176 °C. IR (KBr ν cm^−1^): 3438 (NH), 1677 (C=O), 1289, 1180 (SO_2_). ^1^H-NMR (600 MHz, CDCl_3_) δ ppm 1.24 (s, 3 H), 1.41 (s, 3 H), 3.54 (s, 3 H), 3.66 (d, *J* = 13.75 Hz, 1 H), 3.90 (s, 3 H), 4.44 (d, *J* = 10.09 Hz, 1 H), 4.61 (d, *J* = 9.17 Hz, 1 H), 6.42 (s, 1 H), 7.15 (t, *J* = 8.71 Hz, 2 H), 7.23 (s, 1 H), 8.03 (m, 2 H). ^13^C-NMR (151 MHz, CDCl_3_) δ ppm 23.06, 28.27, 49.89, 55.98, 56.44, 58.13, 58.43, 102.87, 106.74, 116.33, 116.47, 126.89, 132.00, 150.66, 152.78, 165.72, 167.43, 204.13. MS (ESI *m*/*z*) 434.3 [M]^+^. Anal. Calcd. For: C_21_H_23_FN_2_O_5_S: C, 58.05; H, 5.34. Found: C, 58.10; H, 5.30.

*(8,9-Dimethoxy-3,3-dimethyl-6,6-dioxido-2,3,4,10b-tetrahydro-1H-benzo[4,5]isothiazolo[2,3-a]pyrazin-1-yl)(4-Chlorophenyl)methanone* (**14**). Light yellow crystals; yield 39%; m.p. 180–181 °C. IR (KBr ν cm^−1^): 3442 (NH), 1665 (C=O), 1290, 1182 (SO_2_). ^1^H-NMR (600 MHz, CDCl_3_) δ ppm 1.22 (s, 3 H), 1.39 (s, 3 H), 2.93 (d, *J* = 12.84 Hz, 1 H), 3.55 (s, 3 H), 3.66 (d, *J* = 13.75 Hz, 1 H), 3.89 (s, 3 H), 4.41 (d, *J* = 9.17 Hz, 1 H), 4.59 (d, *J* = 9.17 Hz, 1 H), 6.41 (s, 1 H), 7.22 (s, 1 H), 7.44 (d, *J* = 9.17 Hz, 2 H), 7.93 (m, 2 H). ^13^C-NMR (151 MHz, CDCl_3_) δ ppm 23.13, 28.36, 49.53, 49.97, 56.00, 56.43, 58.32, 58.41, 76.89, 77.10, 77.31, 102.86, 106.73, 126.90, 127.06, 129.47, 130.56, 133.87, 141.17, 150.62, 152.78, 197.41. MS (ESI *m*/*z*) 450.2 [M]^+^. Anal. Calcd. For: C_21_H_23_ClN_2_O_5_S: C, 55.94; H, 5.14. Found: C, 55.90; H, 5.20.

*(8,9-Dimethoxy-3,3-dimethyl-6,6-dioxido-2,3,4,10b-tetrahydro-1H-benzo[4,5]isothiazolo[2,3-a]pyrazin-1-yl)(4-bromophenyl)methanone* (**15**). Light yellow crystals; yield 45%; m.p. 195–196 °C. IR (KBr ν cm^−1^): 3447 (NH), 1663 (C=O), 1290, 1182 (SO_2_). ^1^H-NMR (600 MHz, CDCl_3_) δ ppm 1.22 (s, 3 H), 1.39 (s, 3 H), 2.93 (d, *J* = 13.76 Hz, 1 H), 3.55 (s, 3 H), 3.66 (d, *J* = 13.75 Hz, 1 H), 3.90 (s, 3 H), 4.40 (d, *J* = 9.17 Hz, 1 H), 4.59 (d, *J* = 9.17 Hz, 1 H), 6.41 (s, 1 H), 7.22 (s, 1 H), 7.61 (d, *J* = 9.17 Hz, 2 H), 7.84 (m, 2 H). ^13^C-NMR (152 MHz, CDCl_3_) δ ppm 23.13, 28.35, 49.55, 49.97, 56.00, 56.44, 58.31, 58.40, 76.88, 77.10, 77.31, 102.86, 106.73, 126.89, 127.04, 129.98, 130.61, 132.48, 134.26, 150.62, 152.79, 197.63. MS (ESI *m*/*z*) 494.1 [M]^+^. Anal. Calcd. For: C_21_H_23_BrN_2_O_5_S: C, 50.92; H, 4.68. Found: C, 50.95; H, 4.67.

*(8,9-Dimethoxy-3,3-dimethyl-6,6-dioxido-2,3,4,10b-tetrahydro-1H-benzo[4,5]isothiazolo[2,3-a]pyrazin-1-yl)(3-chlorophenyl)methanone* (**16**). Light yellow crystals; yield 54%; m.p. 179–180 °C. IR (KBr ν cm^−1^): 3449 (NH), 1681 (C=O), 1275, 1190 (SO_2_). ^1^H-NMR (600 MHz, CDCl_3_) δ ppm 1.24 (s, 3 H), 1.42 (s, 3 H), 2.95 (d, *J* = 13.75 Hz, 1 H), 3.58 (s, 3 H), 3.67 (d, *J* = 12.84 Hz, 1 H), 3.90 (s, 3 H), 4.41 (d, *J* = 9.17 Hz, 1 H), 4.61 (d, *J* = 9.17 Hz, 1 H), 6.42 (s, 1 H), 7.23 (s, 1 H), 7.42 (t, *J* = 7.79 Hz, 1 H), 7.58 (m, 1 H), 7.84 (d, *J* = 8.25 Hz, 1 H), 7.99 (t, *J* = 1.83 Hz, 1 H). ^13^C-NMR (151 MHz, CDCl_3_) δ ppm 23.05, 28.26, 49.91, 56.06, 56.44, 58.34, 58.48, 102.89, 106.70, 126.89, 127.31, 129.06, 130.43, 134.36, 137.14, 150.66, 152.84. MS (ESI *m*/*z*) 450.2 [M]^+^. Anal. Calcd. For: C_21_H_23_ClN_2_O_5_S: C, 55.94; H, 5.14. Found: C, 56.10; H, 5.23.

*(8,9-Dimethoxy-3,3-dimethyl-6,6-dioxido-2,3,4,10b-tetrahydro-1H-benzo[4,5]isothiazolo[2,3-a]pyrazin-1-yl)(3-bromophenyl)methanone* (**17**). Light yellow crystals; yield 80%; m.p. 175–176 °C. IR (KBr ν cm^−1^): 3447 (NH), 1681 (C=O), 1265, 1190 (SO_2_). ^1^H-NMR (600 MHz, CDCl_3_) δ ppm 1.24 (s, 3 H), 1.42 (s, 3 H), 2.93 (m, 1 H), 3.70 (m, 1 H), 3.87 (s, 3 H), 3.90 (s, 3 H), 4.41 (d, *J* = 9.17 Hz, 1 H), 4.61 (d, *J* = 9.17 Hz, 1 H), 6.42 (s, 1 H), 7.23 (s, 1 H), 7.35 (t, *J* = 7.79 Hz, 1 H), 7.73 (d, *J* = 9.17 Hz, 1 H), 7.87 (d, *J* = 7.34 Hz, 1 H), 8.14 (t, *J* = 1.83 Hz, 1 H). ^13^C-NMR (151 MHz, CDCl_3_) δ ppm 21.84, 23.27, 28.42, 49.30, 50.01, 55.87, 56.40, 57.94, 58.89, 102.75, 106.84, 126.81, 127.22, 129.28, 129.83, 133.25, 145.75, 150.46, 152.64, 198.16. MS (ESI *m*/*z*) 495.9 [M]^+^. Anal. Calcd. For: C_21_H_23_BrN_2_O_5_S: C, 50.92; H, 4.68. Found: C, 50.90; H, 4.60.

*(8,9-Dimethoxy-3,3-dimethyl-6,6-dioxido-2,3,4,10b-tetrahydro-1H-benzo[4,5]isothiazolo[2,3-a]pyrazin-1-yl)(2-chlorophenyl)methanone* (**18**). White crystals; yield 64%; m.p. 191–192 °C. IR (KBr ν cm^−1^): 3448 (NH), 1720 (C=O), 1297, 1179 (SO_2_). ^1^H-NMR (600 MHz, CDCl_3_) δ ppm 1.43 (s, 3 H), 1.57 (s, 3 H), 2.96 (dd, *J* = 16.05, 11.46 Hz, 1 H), 3.20 (d, *J* = 13.75 Hz, 1 H), 3.39 (m, 1 H), 3.67 (d, *J* = 14.67 Hz, 1 H), 3.92 (s, 3 H), 3.94 (s, 3 H), 4.63 (d, *J* = 11.00 Hz, 1 H), 6.75 (s, 1 H), 7.23 (s, 1 H), 7.32 (d, *J* = 2.75 Hz, 3 H), 7.40 (m, 1 H). ^13^C-NMR (151 MHz, CDCl_3_) δ ppm 23.26, 31.34, 42.19, 50.76, 56.54, 58.60, 60.05, 102.91, 104.78, 125.99, 127.55, 129.32, 129.70, 129.87, 130.01, 130.95, 143.44, 150.77, 153.75, 164.95. MS (ESI *m*/*z*) 450.2 [M]^+^. Anal. Calcd. For: C_21_H_23_ClN_2_O_5_S: C, 55.94; H, 5.14. Found: C, 55.92; H, 5.17.

*(8,9-Dimethoxy-3,3-dimethyl-6,6-dioxido-2,3,4,10b-tetrahydro-1H-benzo[4,5]isothiazolo[2,3-a]pyrazin-1-yl)(2-bromophenyl)methanone* (**19**). White crystals; yield 29%; m.p. 201–202 °C. IR (KBr ν cm^−1^): 3467 (NH), 1715 (C=O), 1297, 1179 (SO_2_). ^1^H-NMR (600 MHz, CDCl_3_) δ ppm 1.43 (s, 3 H), 1.57 (s, 3 H), 2.95 (dd, *J* = 15.59, 11.92 Hz, 1 H), 3.21 (d, *J* = 13.75 Hz, 1 H), 3.36 (m, 1 H), 3.67 (d, *J* = 14.67 Hz, 1 H), 3.92 (s, 3 H), 3.93 (s, 3 H), 4.72 (d, *J* = 11.00 Hz, 1 H), 6.78 (s, 1 H), 7.23 (s, 1 H), 7.25 (m, 1 H), 7.29 (m, 1 H), 7.36 (m, 1 H), 7.59 (d, *J* = 8.25 Hz, 1 H). ^13^C-NMR (151 MHz, CDCl_3_) δ ppm 23.49, 31.11, 42.25, 50.81, 56.53, 58.88, 60.09, 76.88, 77.09, 77.30, 102.91, 104.76, 120.07, 125.96, 128.09, 129.33, 129.70, 130.09, 132.97, 145.30, 150.77, 153.77, 165.54. MS (ESI *m*/*z*) 498.6 [M]^+^. Anal. Calcd. For: C_21_H_23_BrN_2_O_5_S: C, 50.92; H, 4.68. Found: C, 50.85; H, 4.75.

Supplementary materials: ^1^H- and ^13^C-NMR spectra for compounds **2** to **19**; X-ray data for compound **11**.

### 3.7. Antimicrobial Activity

The benzo[4,5]isothiazolo[2,3-*a*]pyrazine-6,6-dioxide derivatives **2**–**19** were screened against four bacteria, *B. subtilis* ATCC 6633, *E. coli* ATCC 25922, *P. vulgaris* ATCC 13315 (ATCC 29905) and *S. aureus* ATCC 6538. The broth micro dilution method (with a slight modification as described by the Clinical and Laboratory Standards Institute) using 96-well micro-titre plates was used to test the antibacterial activity of the compounds **2**–**19** [29]. The concentration of the compounds was tested in a range of 6.67 mg·mL^−1^ down to 0.052 mg·mL^−1^. Dilutions were carried out so that each well had 50 μL of Mueller–Hinton broth with a particular concentration of the compound. An equal volume of bacterial culture adjusted to 0.5 McFarland turbidity standard and further diluted to 1:20 was added to the wells and incubated at 37 °C for 16–18 h. Plates were then examined for turbidity as an indicator of growth, and the absorbance was taken using an ELISA reader at 590 nm [30]. The Minimum Inhibitiry Concentration (MIC) of the test compounds were further confirmed by adding a concentration of 5 mg/mL MTT [3-(4,5-dimethylthiazol-2-yl)-2,5-diphenyltetrazolium bromide] was prepared in Phosphate Buffered Saline (PBS) (pH 7.2). Twenty μL of MTT solution was added to each single well and the 96-well micro-titer plates were incubated for 30 min at 30 °C. The wells with lowest concentration of the compound where there was no formazan were confirmed as the MIC of the test compounds.

Streptomycin was used as a reference drug throughout all the antibacterial testing.

## 4. Conclusions

Using a routine procedure, the synthesis of a new series of benzo[4,5]isothiazolo[2,3-*a*]pyrazine-6,6-dioxide derivatives starting from readily available chalcones was carried out. This methodology is straightforward and the isolation of desired products is simple. Unfortunately, the synthesized compounds displayed only weak or no antibacterial activity.

## Figures and Tables

**Table 1:** Data for benzo[4,5]isothiazolo[2,3-*a*]pyrazine-6,6-dioxide derivatives **2**–**19**.

Compound	R^1^	R^2^	R^3^	R^4^	Yield (%)	m.p. (°C)	*m*/*z* (M^+^)
**2**	H	H	H	H	29	219–220	402.3
**3**	CH_3_	H	H	H	47	180–181	416.3
**4**	F	H	H	H	30	181–182	420.2
**5**	Cl	H	H	H	43	204–205	436.2
**6**	Br	H	H	H	48	204–205	480.4
**7**	H	Cl	H	H	79	194–195	436.2
**8**	H	Br	H	H	50	189–190	482.7
**9**	H	H	Cl	H	46	215–216	436.5
**10**	H	H	Br	H	75	195–196	480.4
**11**	H	H	H	CH_3_	34	163–164	416.3
**12**	CH_3_	H	H	CH_3_	64	205–206	430.3
**13**	F	H	H	CH_3_	12	175–176	434.3
**14**	Cl	H	H	CH_3_	39	180–181	450.2
**15**	Br	H	H	CH_3_	45	195–196	494.1
**16**	H	Cl	H	CH_3_	54	179–180	450.2
**17**	H	Br	H	CH_3_	80	175–176	495.9
**18**	H	H	Cl	CH_3_	64	191–192	450.2
**19**	H	H	Br	CH_3_	29	201–202	498.6

**Table 2:** MIC (μg/mL) of the compounds **2**–**19** against Gram Positive bacteria *Bacillus. subtilis* and *S* *Staphylococcus* *aureus* and Gram negative bacteria *Proteus* *vulgaris* and *Escherichia* *coli*.

Compound	*S. aureus* (MIC μg/mL)	*B. subtilis* (MIC μg/mL)	*E. coli* (MIC μg/mL)	*P. vulgaris* (MIC μg/mL)
**2**	>1600	>1600	>1600	>1600
**3**	>1600	>1600	>1600	>1600
**4**	>1600	>1600	>1600	>1600
**5**	>1600	>1600	>1600	>1600
**6**	>1600	>1600	>1600	>1600
**7**	1600	833	1600	1600
**8**	>1600	>1600	>1600	>1600
**9**	>1600	>1600	>1600	>1600
**10**	>1600	>1600	>1600	>1600
**11**	>1600	>1600	>1600	>1600
**12**	>1600	>1600	>1600	>1600
**13**	>1600	>1600	>1600	>1600
**14**	>1600	>1600	>1600	>1600
**15**	>1600	>1600	>1600	>1600
**16**	>1600	>1600	>1600	>1600
**17**	>1600	>1600	>1600	>1600
**18**	>1600	>1600	>1600	>1600
**19**	>1600	>1600	>1600	>1600
**Streptomycin**	12.5	12.5	12.5	6.25

## Supplementary Materials

Supplementary materials are available online (^1^H- and ^13^C-NMR spectra for compounds **2** to **19**; X-ray data for compound **11**).
