# Supplementary material for: Synthesis and Antibacterial Activity of Benzo[4,5]isothiazolo[2,3-a]pyrazine-6,6-dioxide Derivatives
Source: Molecules. 2017 Nov 4;22(11):1889. doi: 10.3390/molecules22111889 (PMC6150388; doi:10.3390/molecules22111889)
Supplement: Supplementary file 1 [file molecules-22-01889-s001.pdf]

Supplementary Material

Synthesis and antibacterial activity of benzo[4,5]isothiazolo[2,3-a]pyrazine-6,6-dioxide derivatives

Jatinder P. Bassin[1]*, Michelle J. Botha[1], Rajesh Garikipati[1], Madhu Goyal[1]*, Lee Martin[2] and Amit Shah[1]

1 School of Life and Medical Sciences, University of Hertfordshire, Hatfield, AL10 9AB, United Kingdom ; m.botha@herts.ac.uk (M.J.B.) ; r.garikipati4@gmail.com (R.G.) and amit_pharmacy@hotmail.co.uk (A.S.).

2 School of Science and Technology, Nottingham Trent University, Clifton Lane, Clifton, Nottingham NG11 8NS, UK; lee.martin@ntu.ac.uk.

* Correspondence: j.p.bassin@herts.ac.uk (J.P.B.); m.goyal@herts.ac.uk (M.G.);
Tel.: +44-1707-285097 (J.P.B.); +44-1707-284624 (M.G.)

Table of Content

	Page
Proton NMR for compound **2**	S3
Carbon NMR for compound **2**	S4
Proton NMR for compound **3**	S5
Carbon NMR for compound **3**	S6
Proton NMR for compound **4**	S7
Carbon NMR for compound **4**	S8
Proton NMR for compound **5**	S9
Carbon NMR for compound **5**	S10
Proton NMR for compound **6**	S11
Carbon NMR for compound **6**	S12
Proton NMR for compound **7**	S13
Carbon NMR for compound **7**	S14
Proton NMR for compound **8**	S15
Carbon NMR for compound **8**	S16
Proton NMR for compound **9**	S17
Carbon NMR for compound **9**	S18
Proton NMR for compound **10**	S19
Carbon NMR for compound **10**	S20
Proton NMR for compound **11**	S21
Carbon NMR for compound **11**	S22
Proton NMR for compound **12**	S23
Carbon NMR for compound **12**	S24
Proton NMR for compound **13**	S25
Carbon NMR for compound **13**	S26
Proton NMR for compound **14**	S27
Carbon NMR for compound **14**	S28
Proton NMR for compound **15**	S29
Carbon NMR for compound **15**	S30
Proton NMR for compound **16**	S31
Carbon NMR for compound **16**	S32
Proton NMR for compound **17**	S33
Carbon NMR for compound **17**	S34
Proton NMR for compound **18**	S35
Carbon NMR for compound **18**	S36
Proton NMR for compound **19**	S37
Carbon NMR for compound **19**	S38
Crystal data and structure refinement for compound **11**.	S39
Table i	S40
Table ii	S41
Table iii	S44
Table iv	S45
Table v	S45

Proton NMR for compound 2

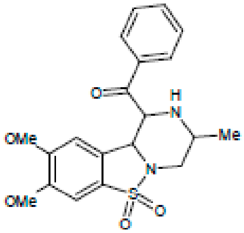

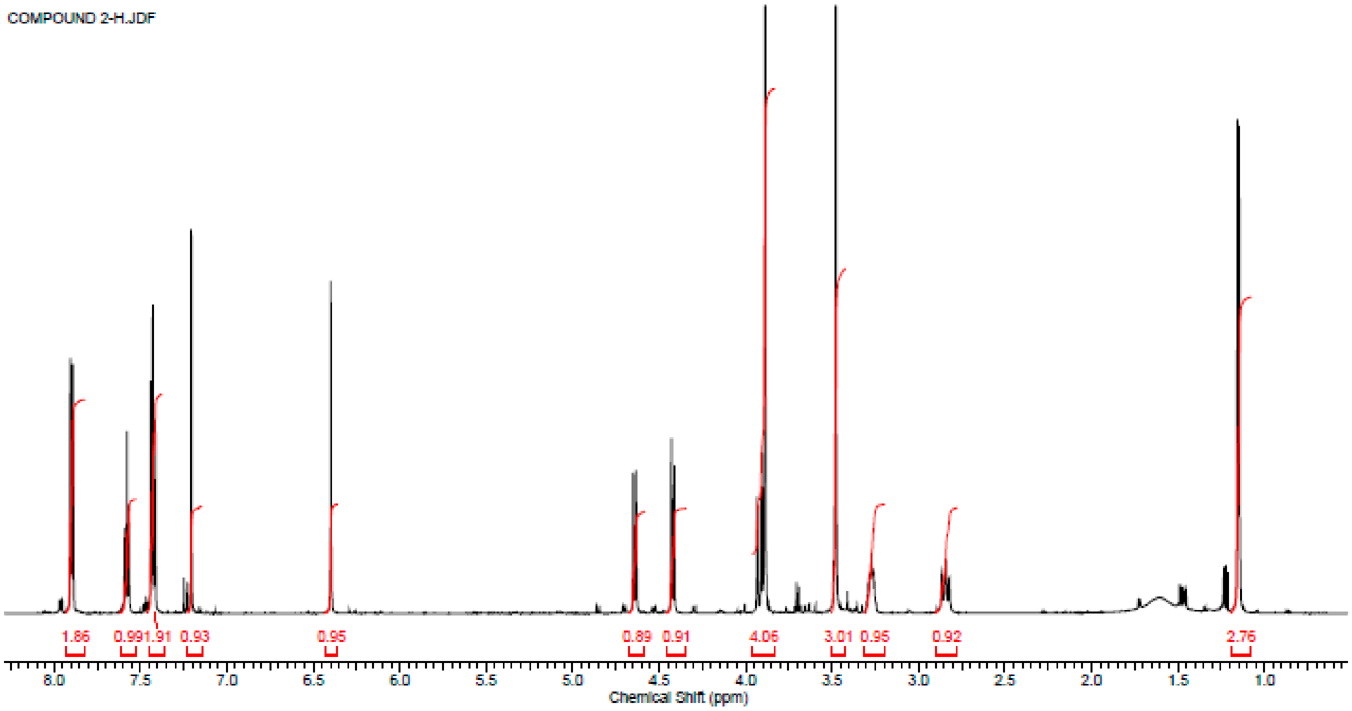

Carbon NMR for compound 2

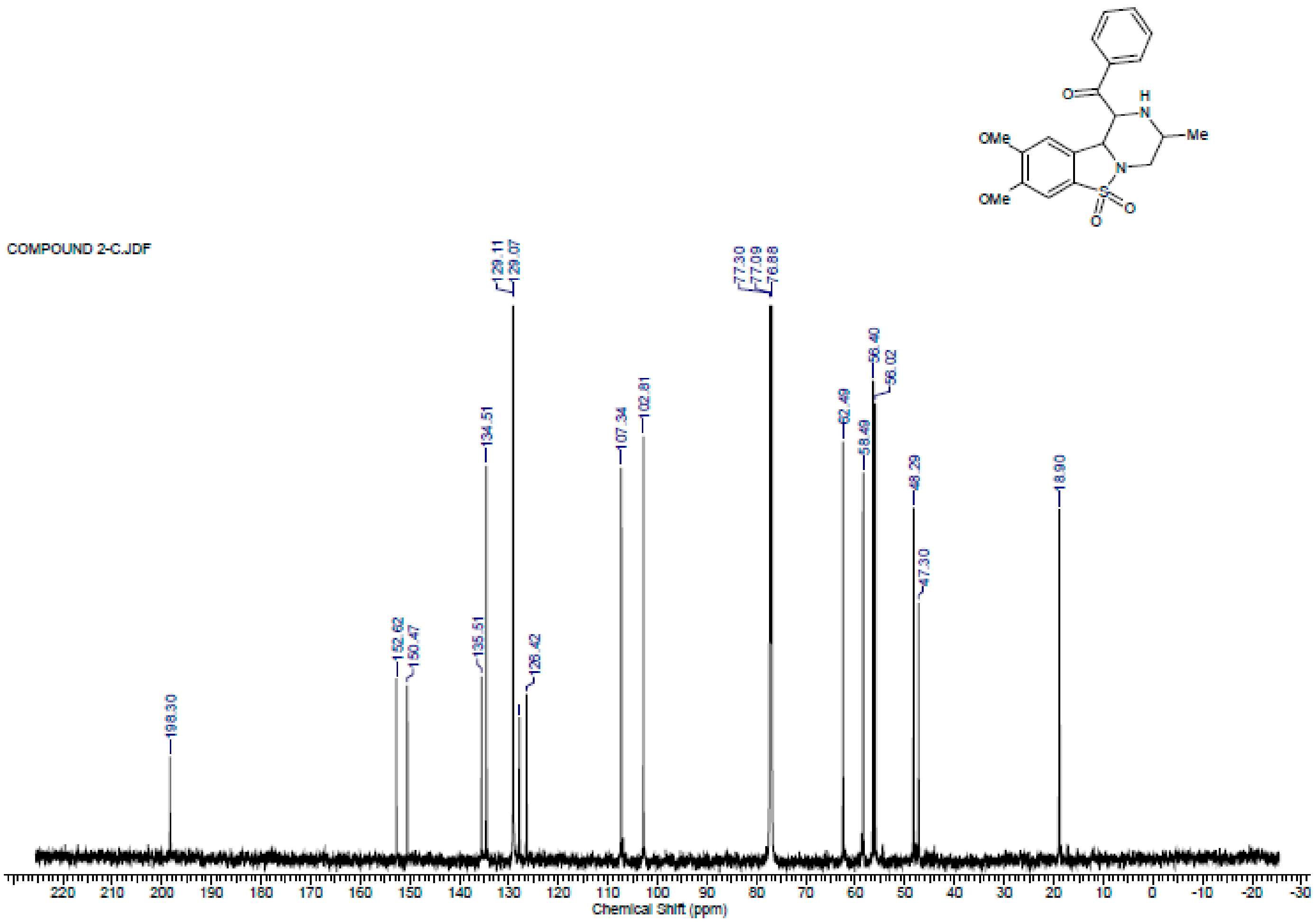

Proton NMR for compound 3

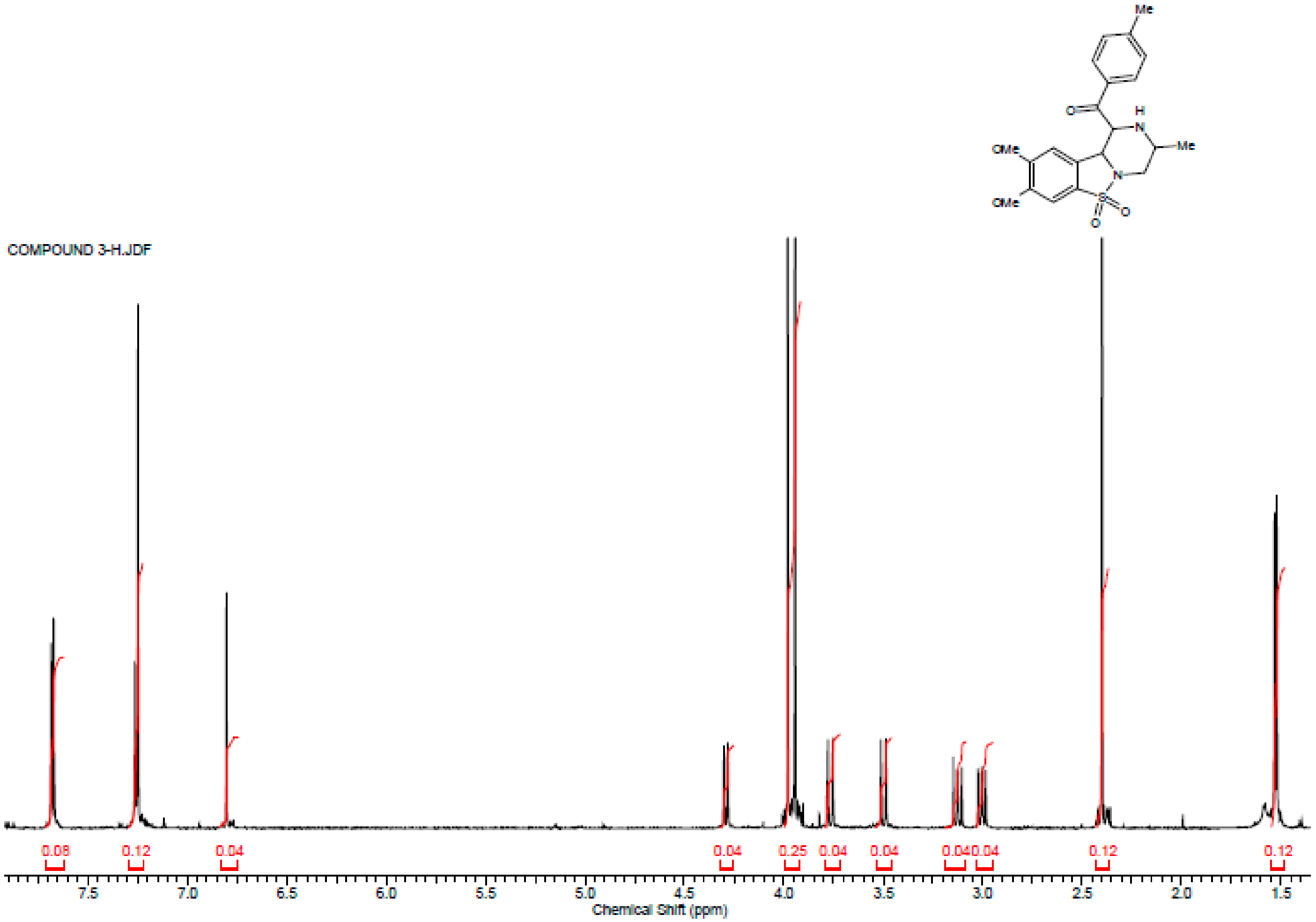

Carbon NMR for compound 3

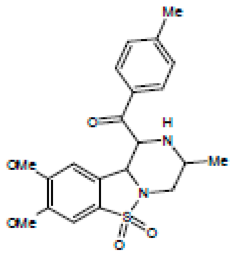

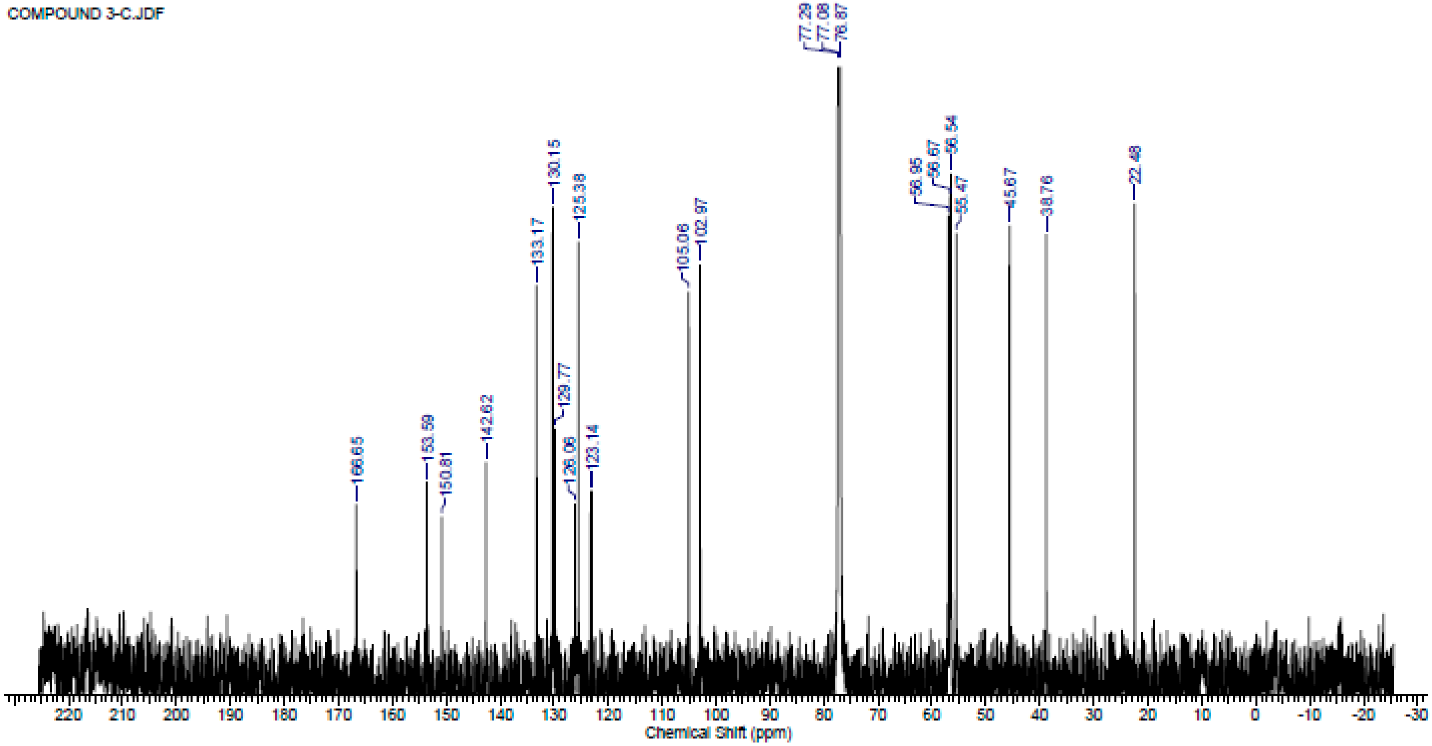

Proton NMR for compound 4

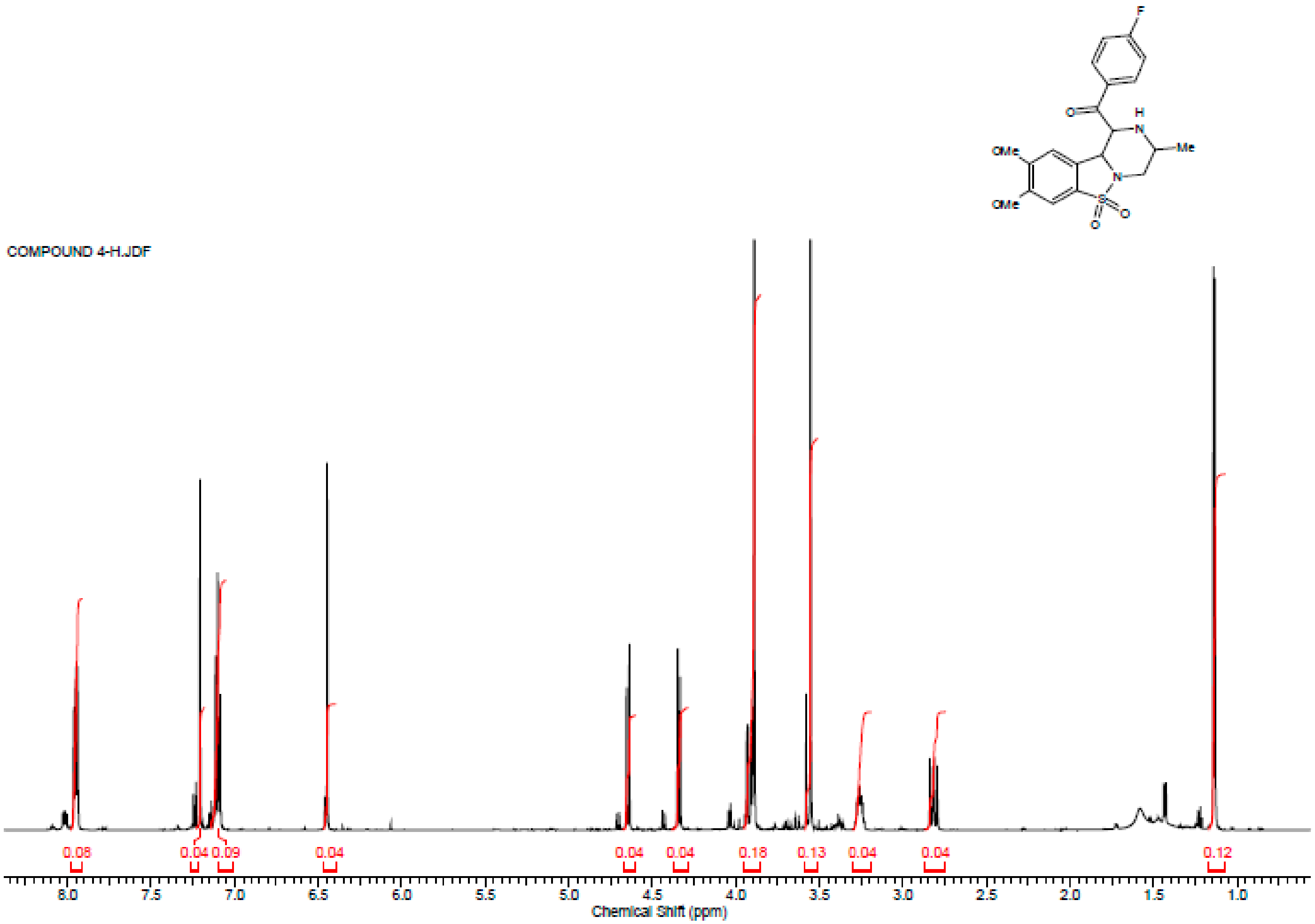

Carbon NMR for compound 4

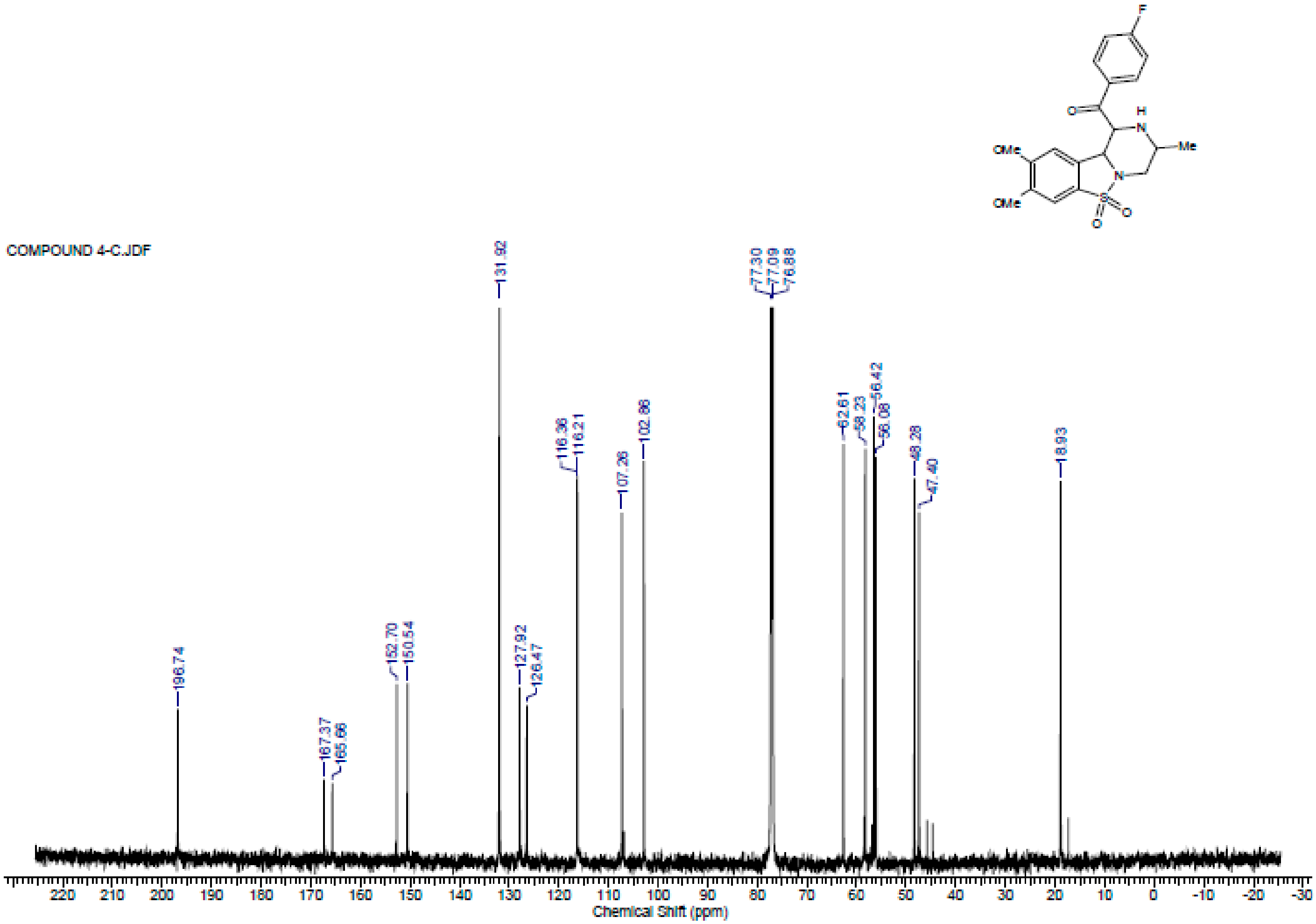

Proton NMR for compound 5

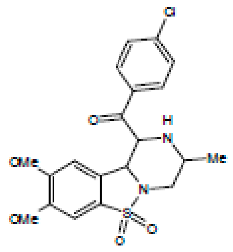

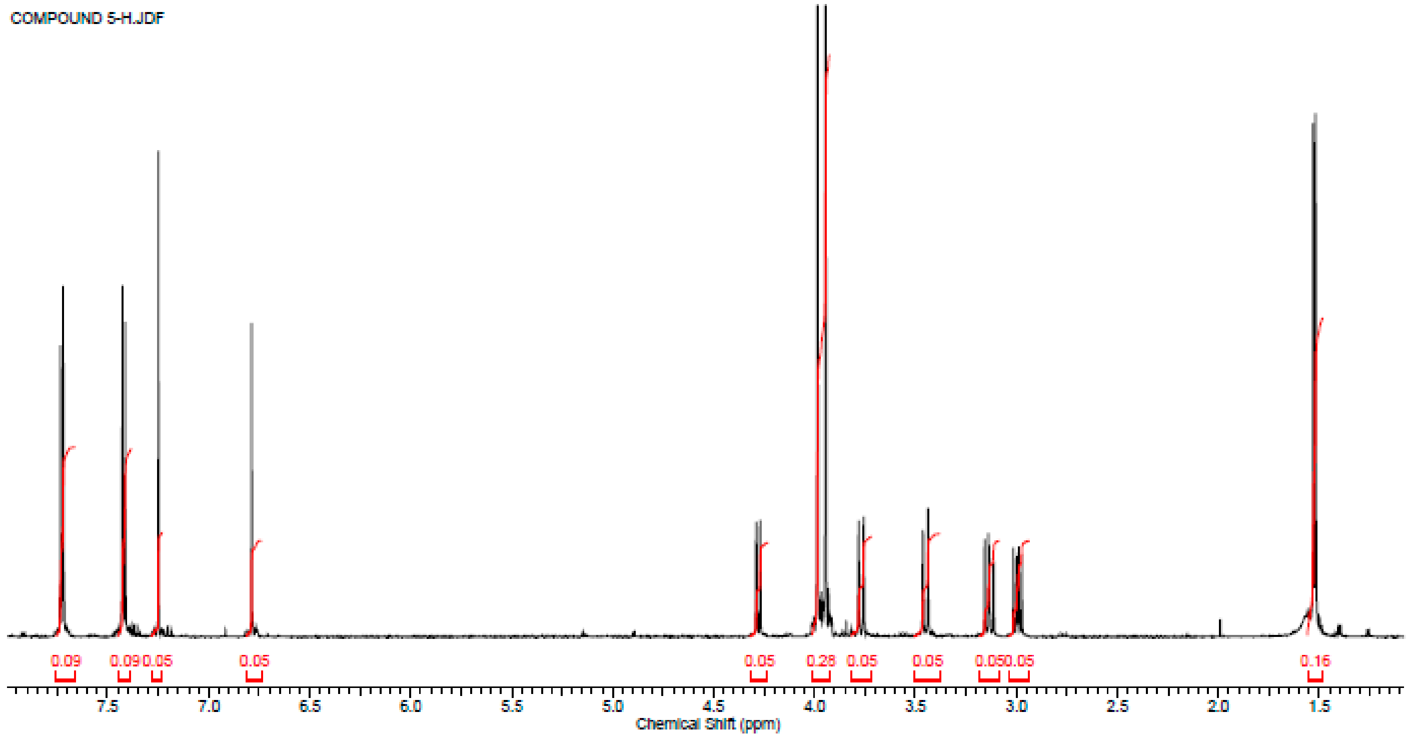

Carbon NMR for compound 5

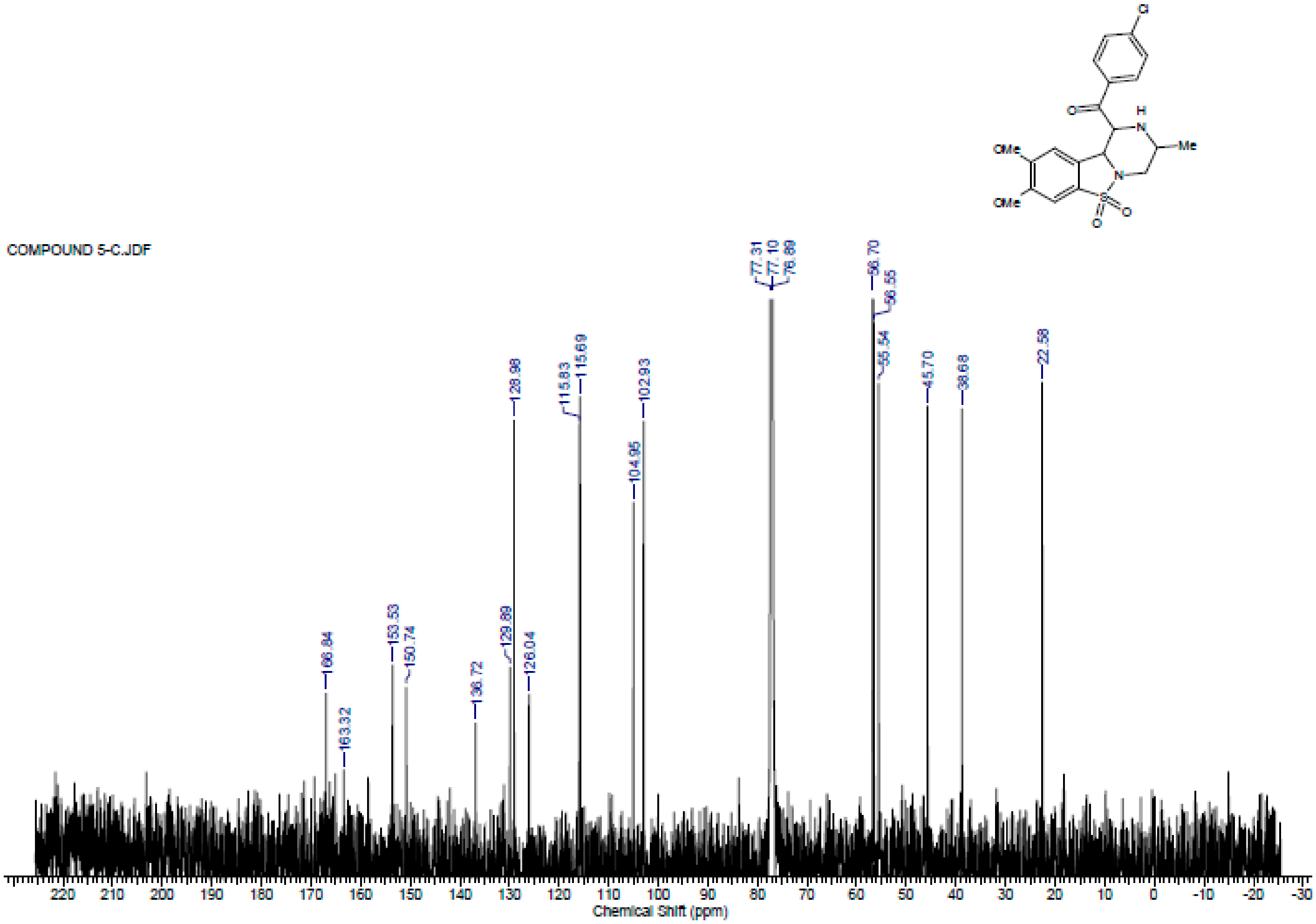

Proton NMR for compound 6

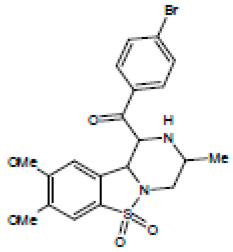

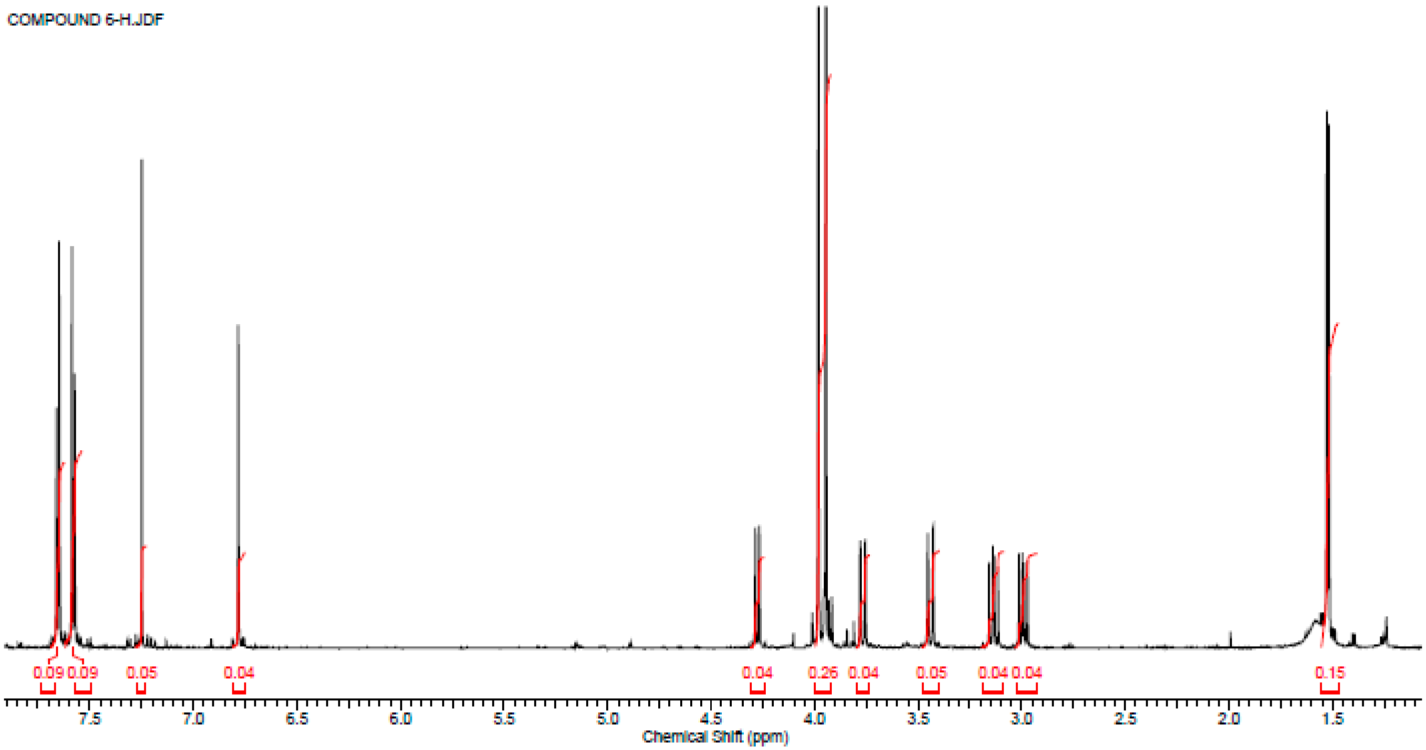

Carbon NMR for compound 6

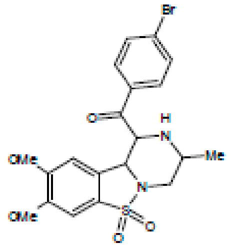

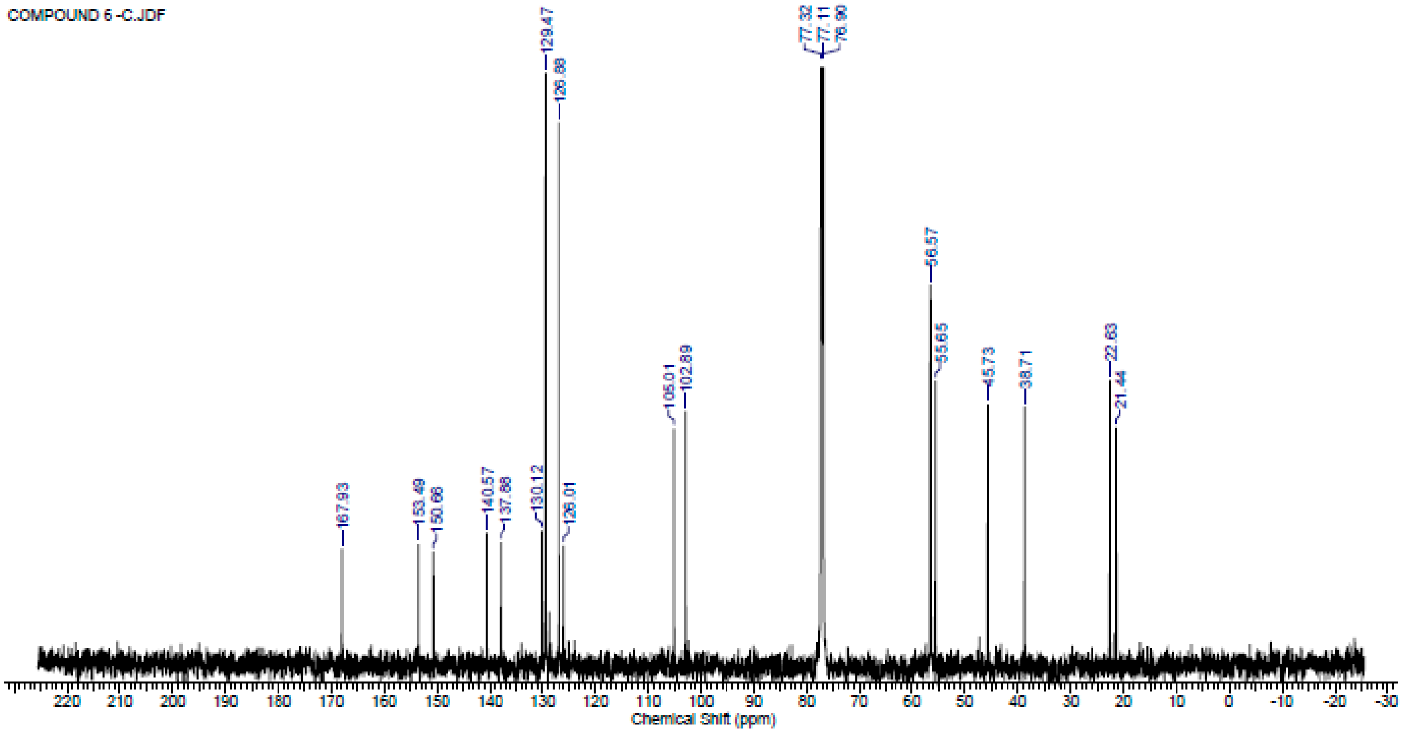

Proton NMR for compound 7

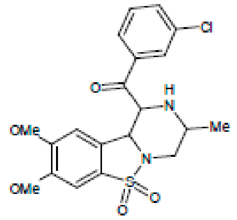

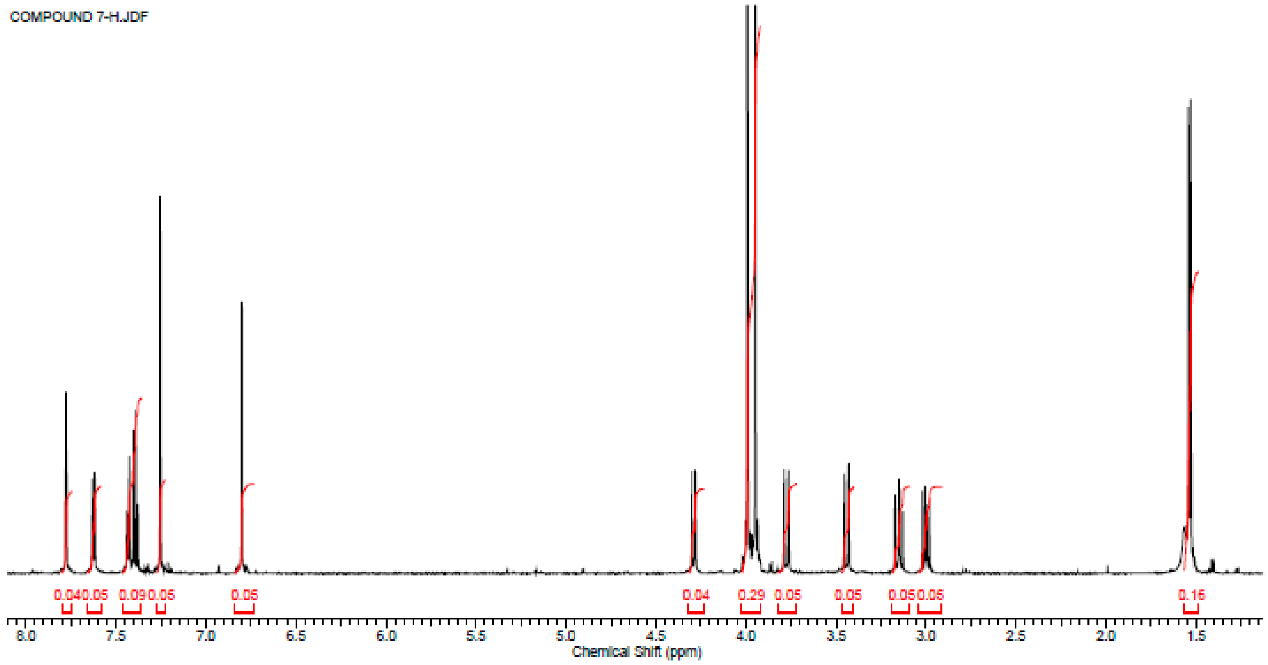

Carbon NMR for compound 7

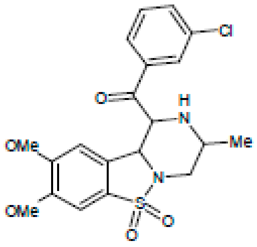

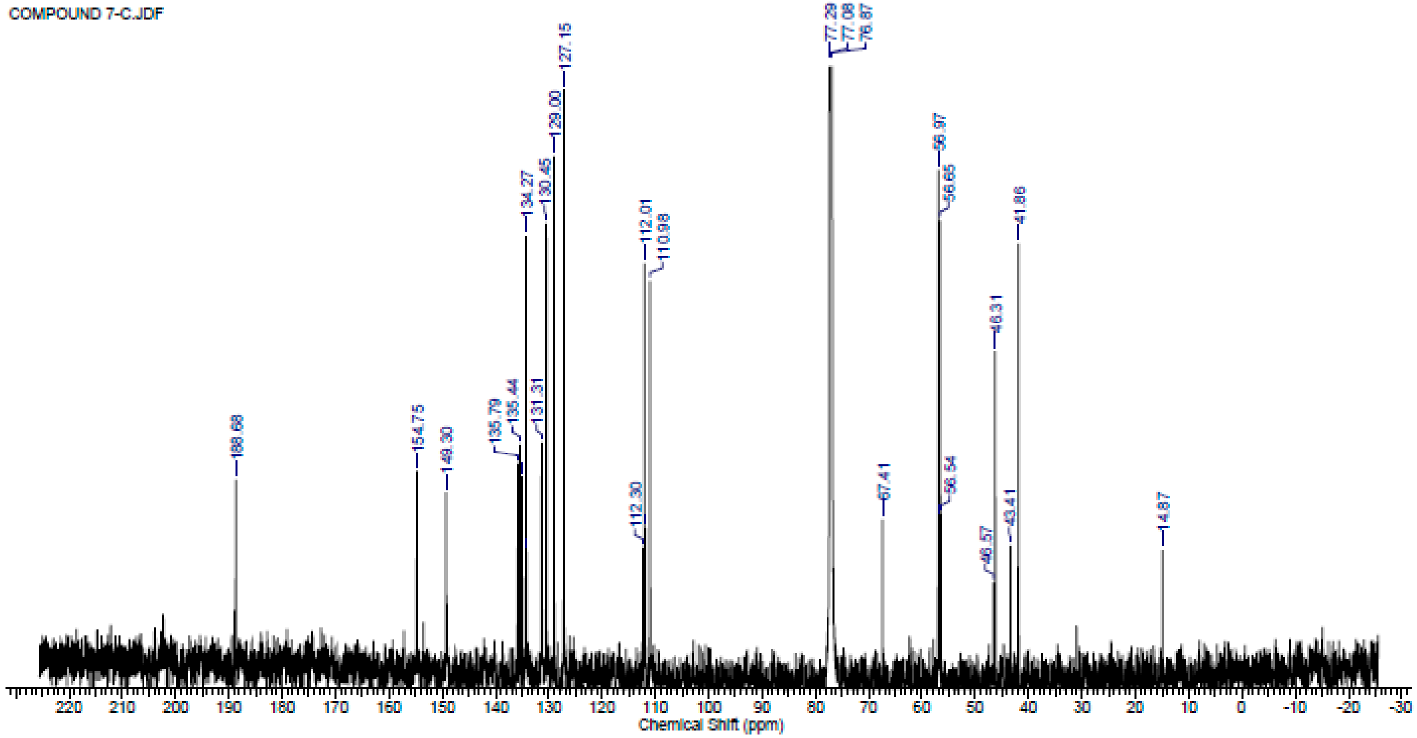

Proton NMR for compound 8

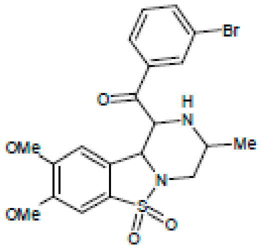

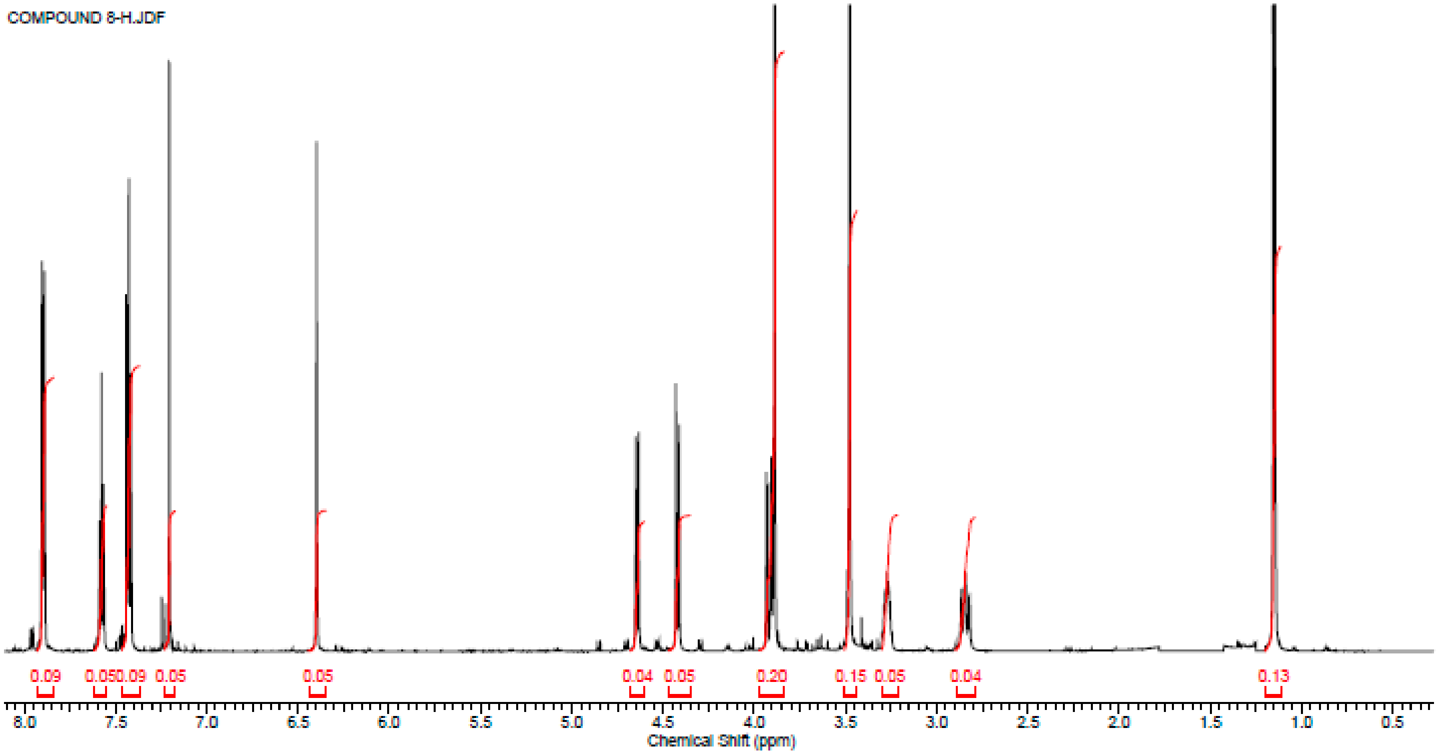

Carbon NMR for compound 8

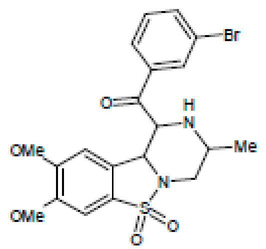

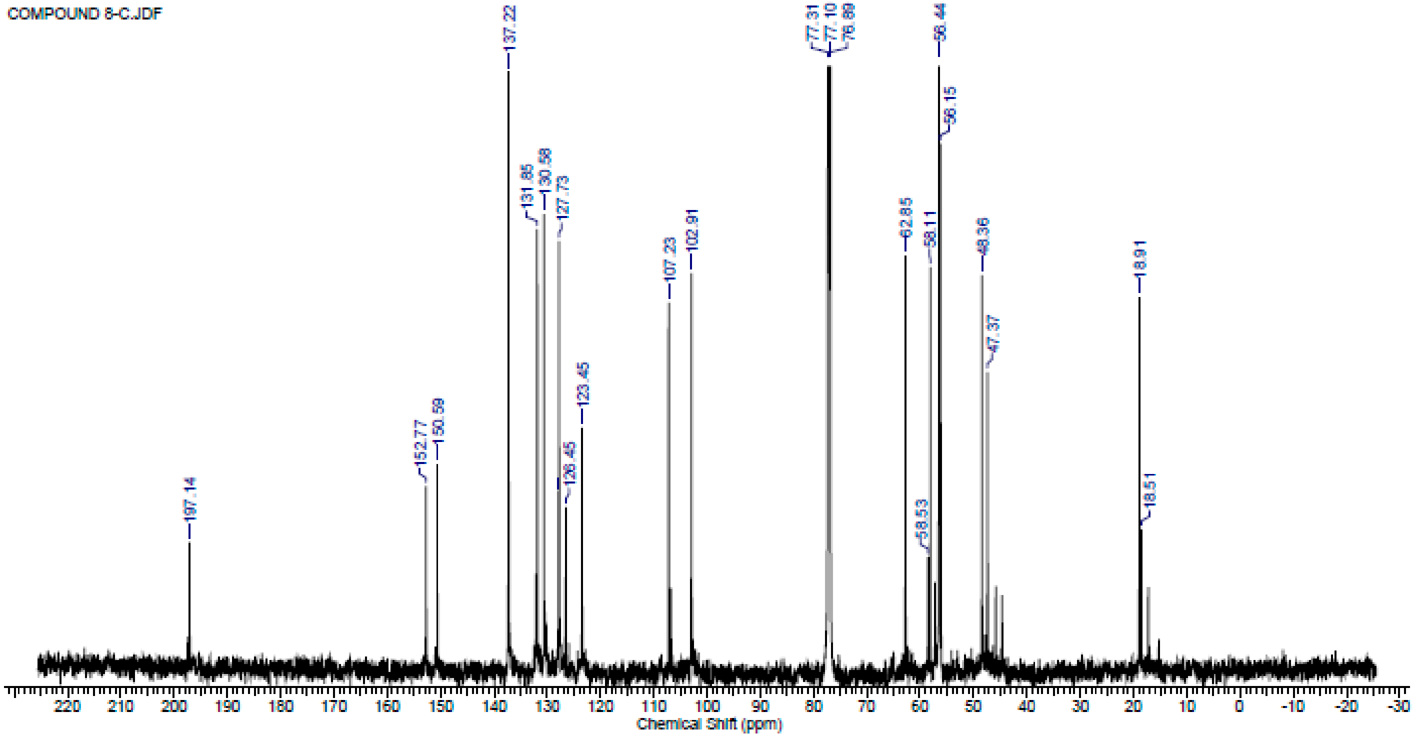

Proton NMR for compound 9

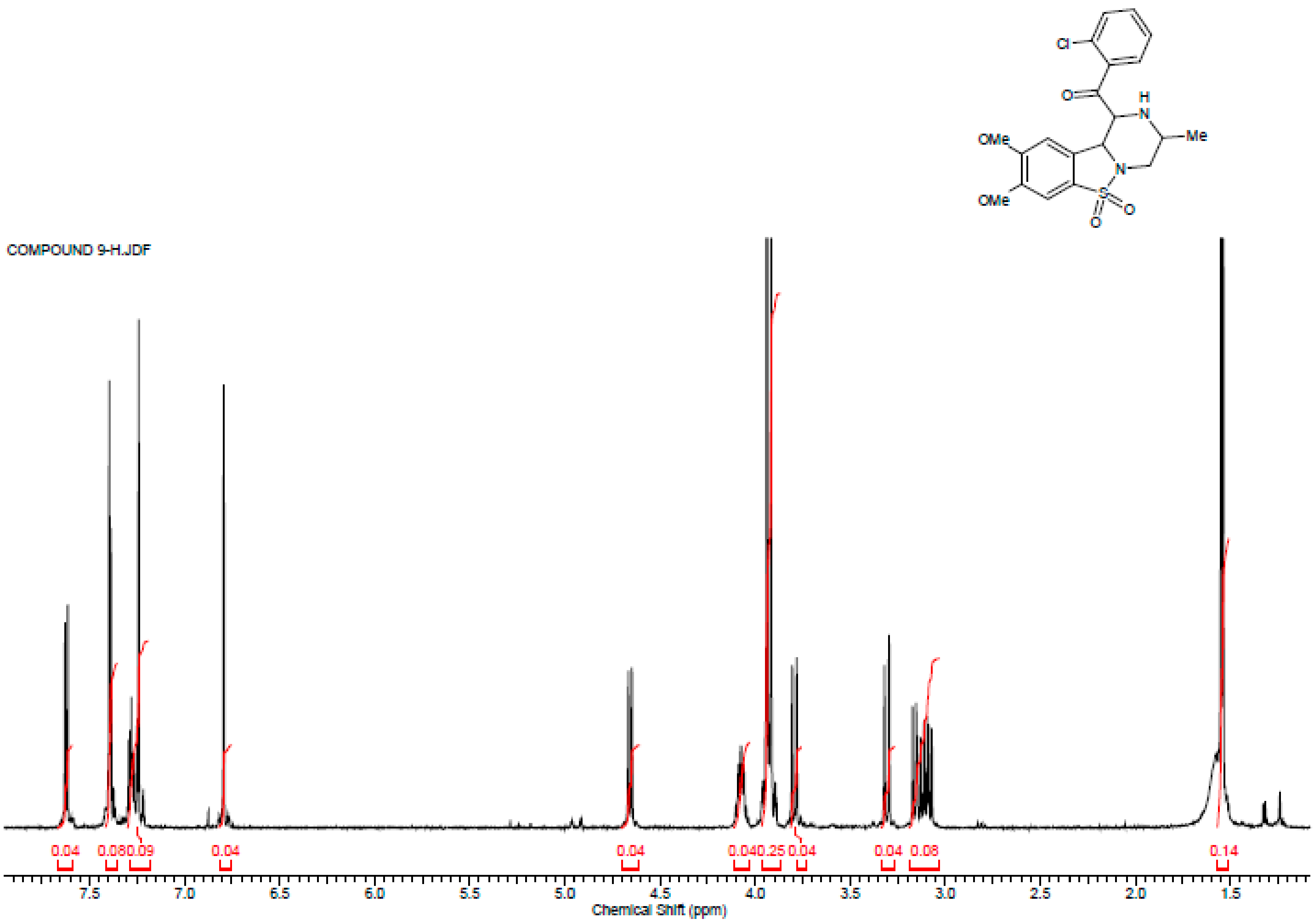

Carbon NMR for compound 9

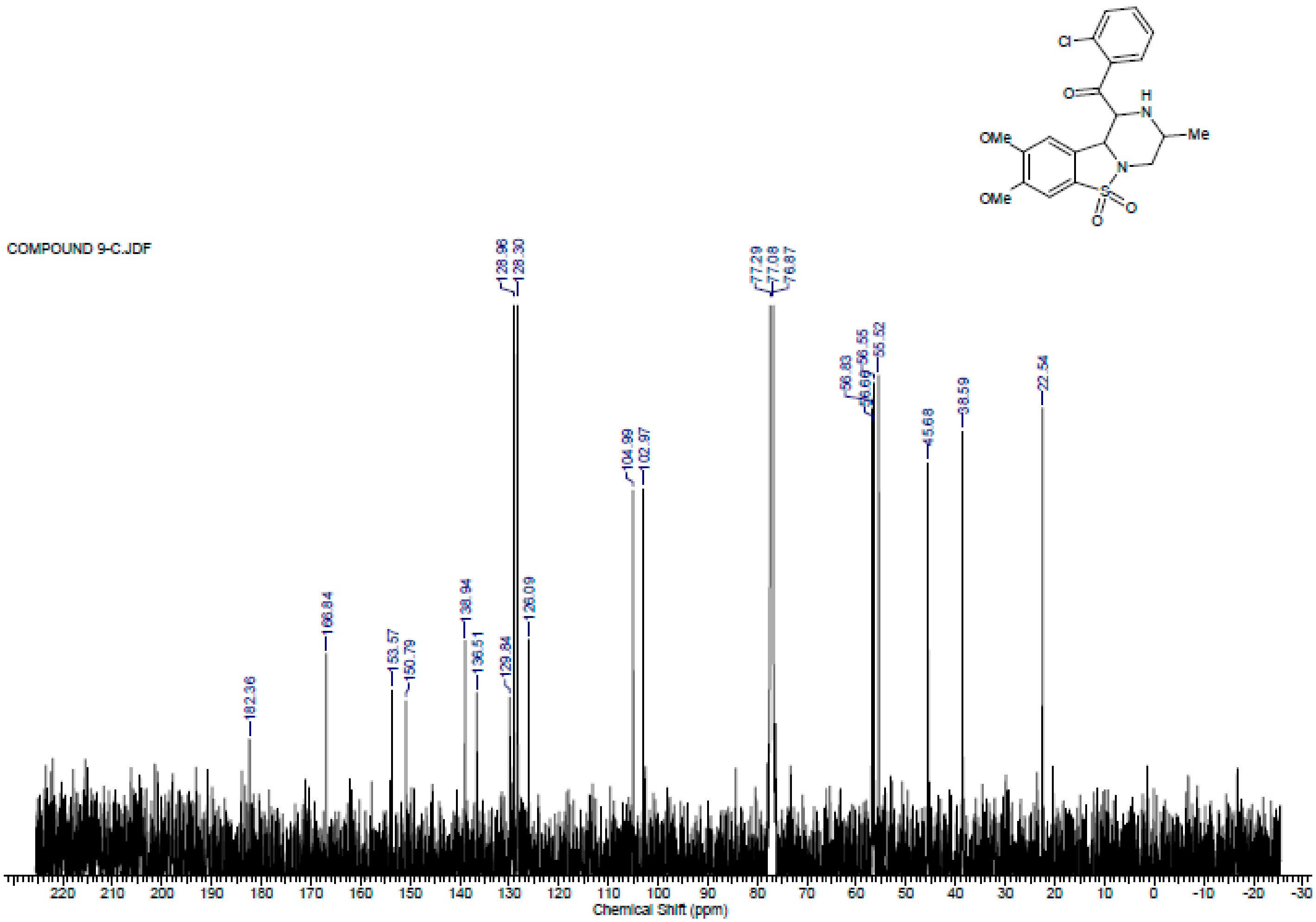

Proton NMR for compound 10

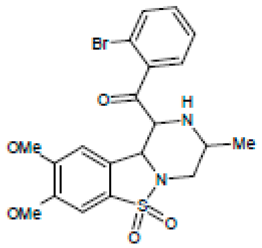

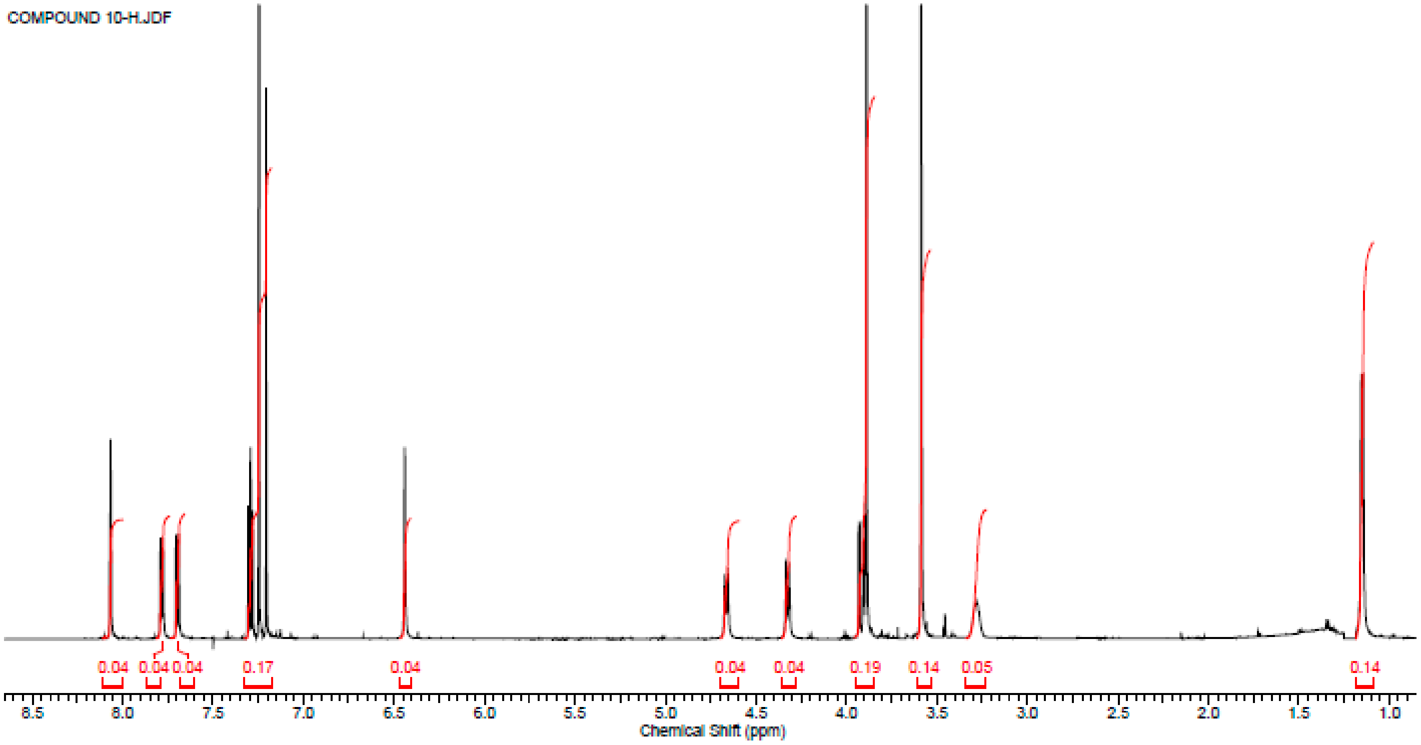

Carbon NMR for compound 10

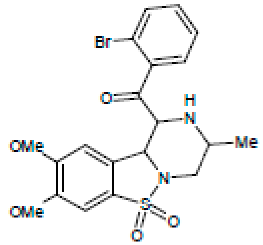

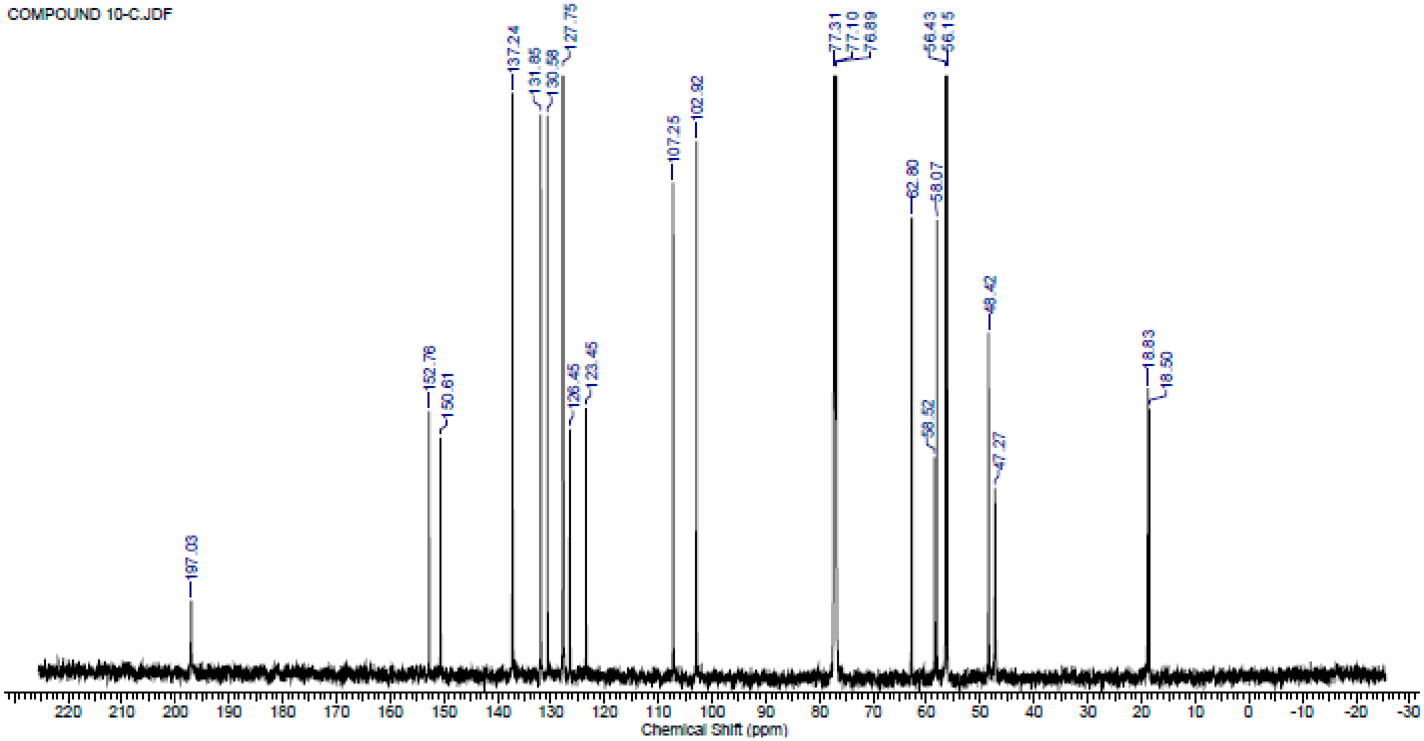

Proton NMR for compound 11

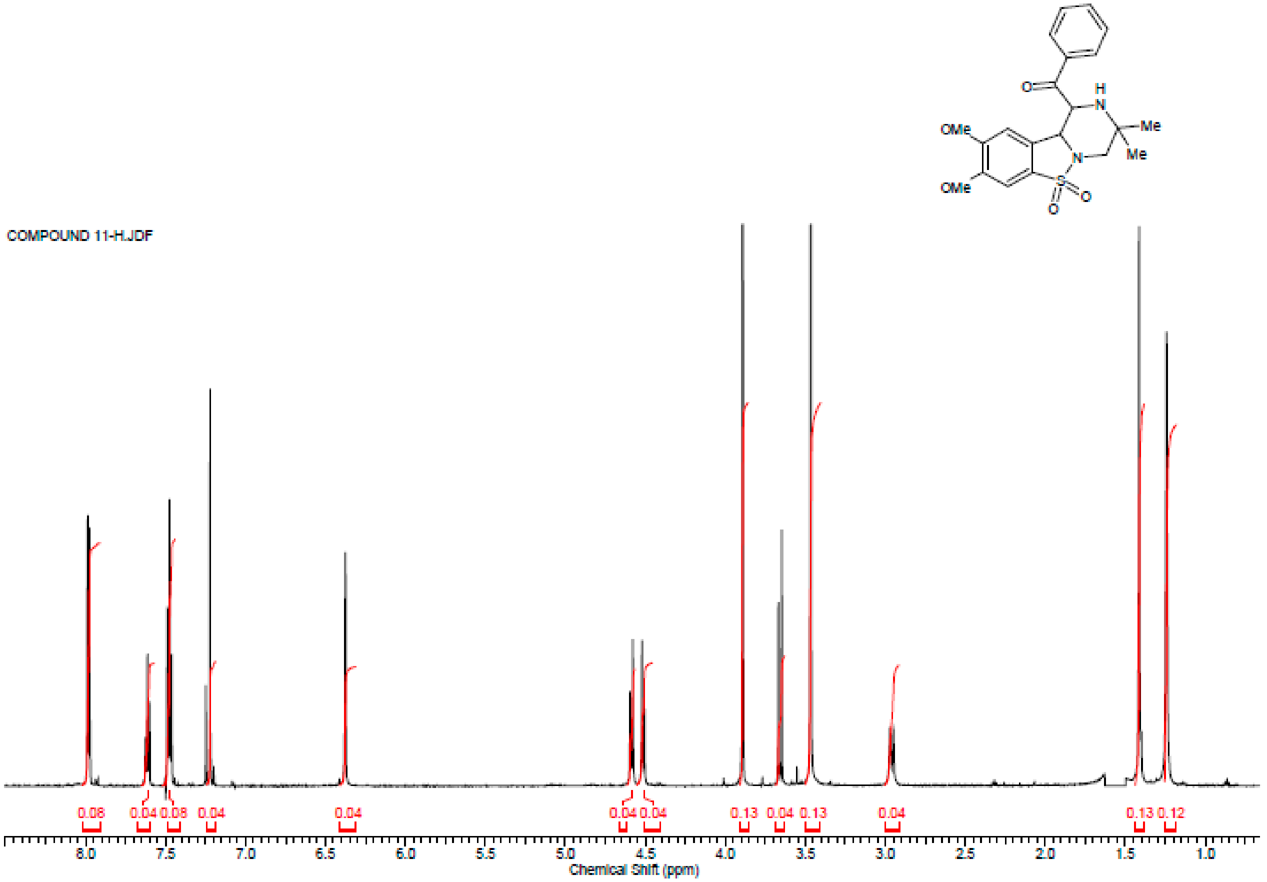

Carbon NMR for compound 11

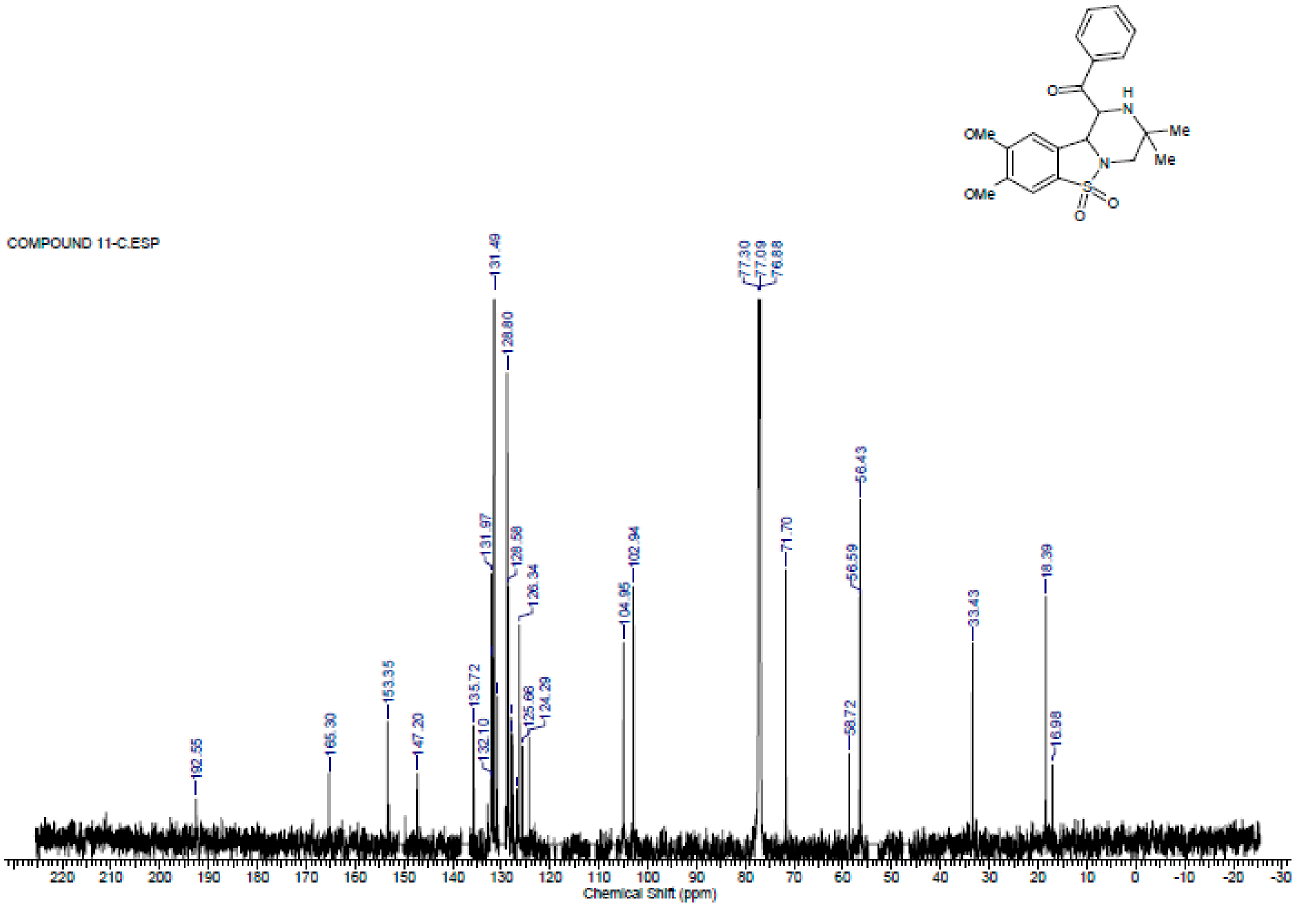

Proton NMR for compound 12

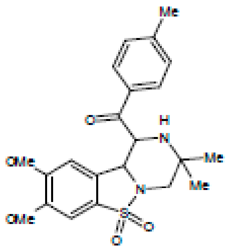

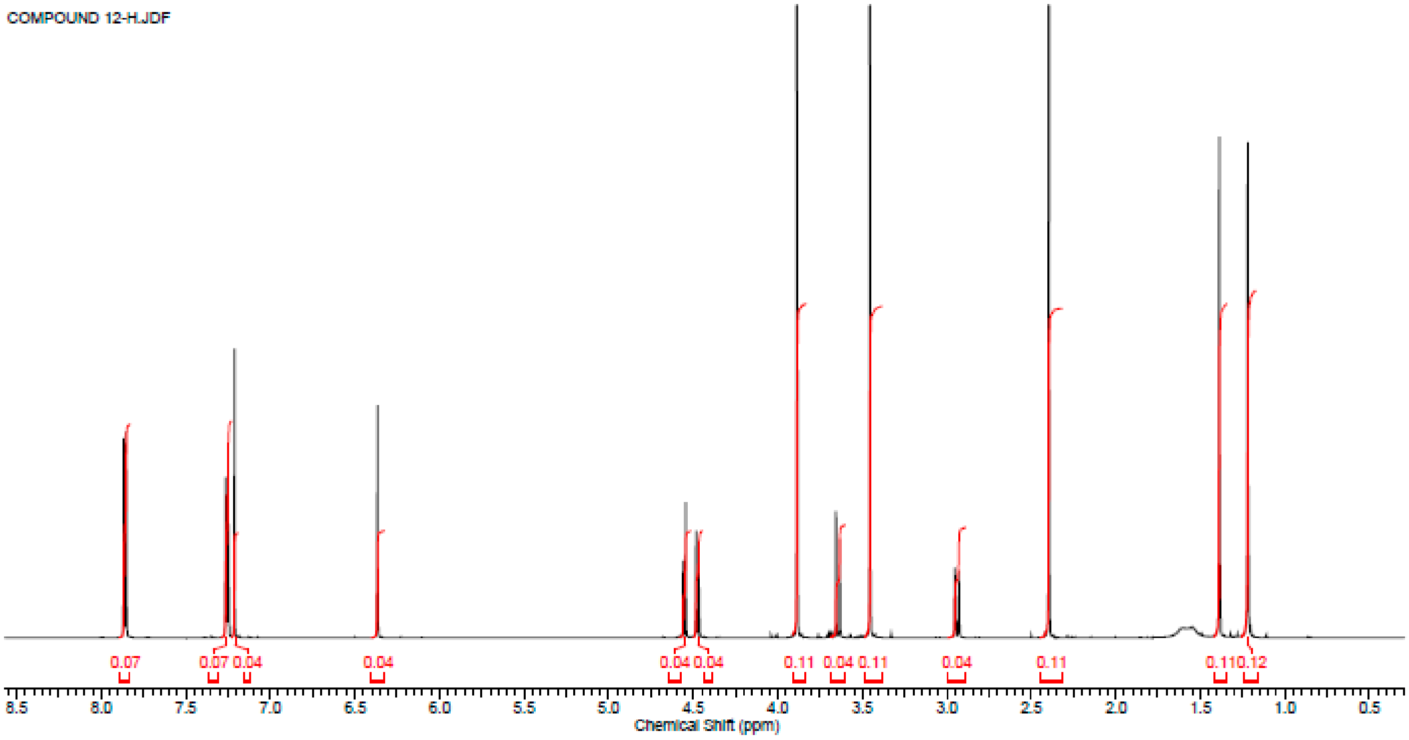

Carbon NMR for compound 12

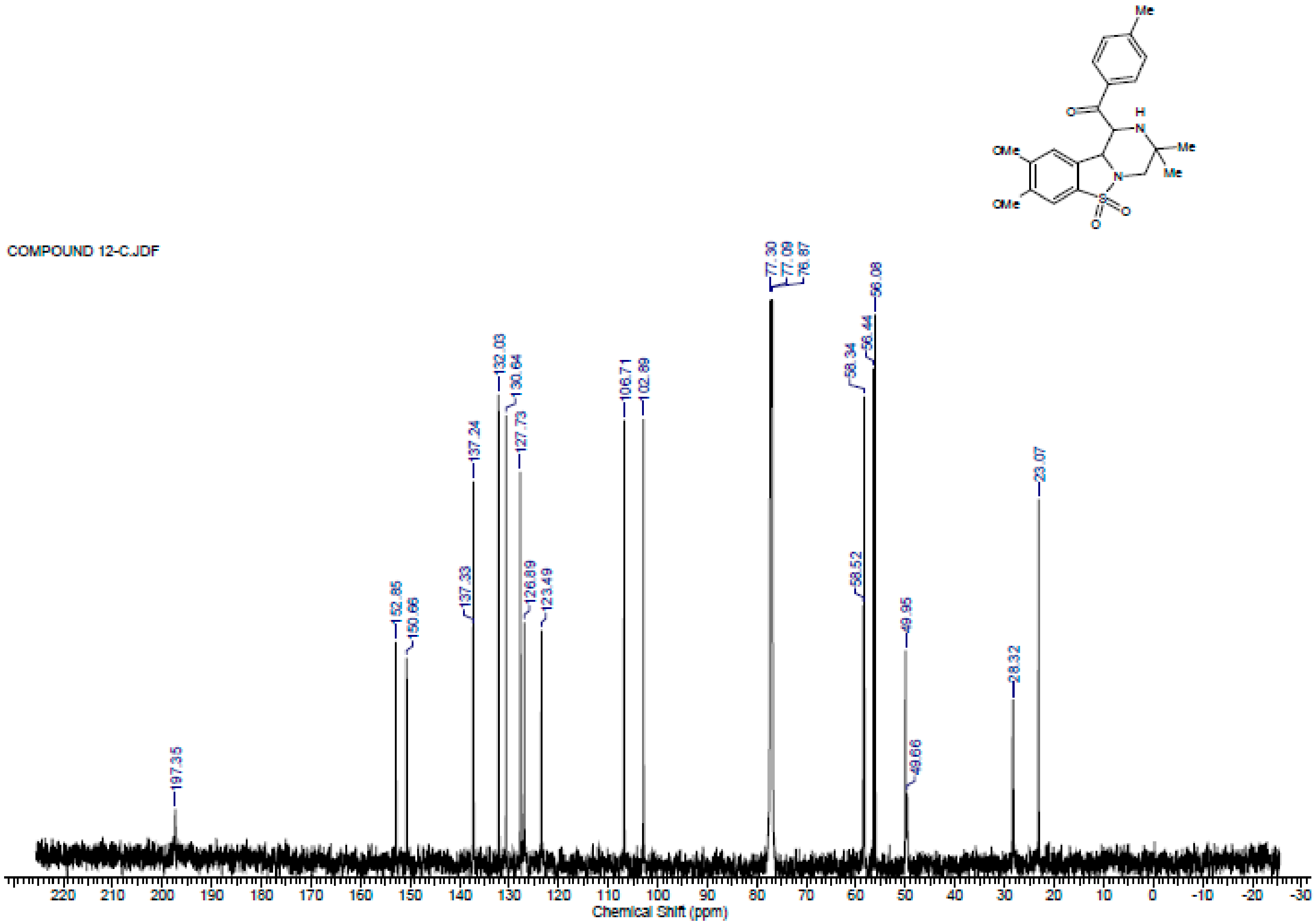

Proton NMR for compound 13

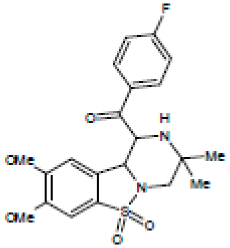

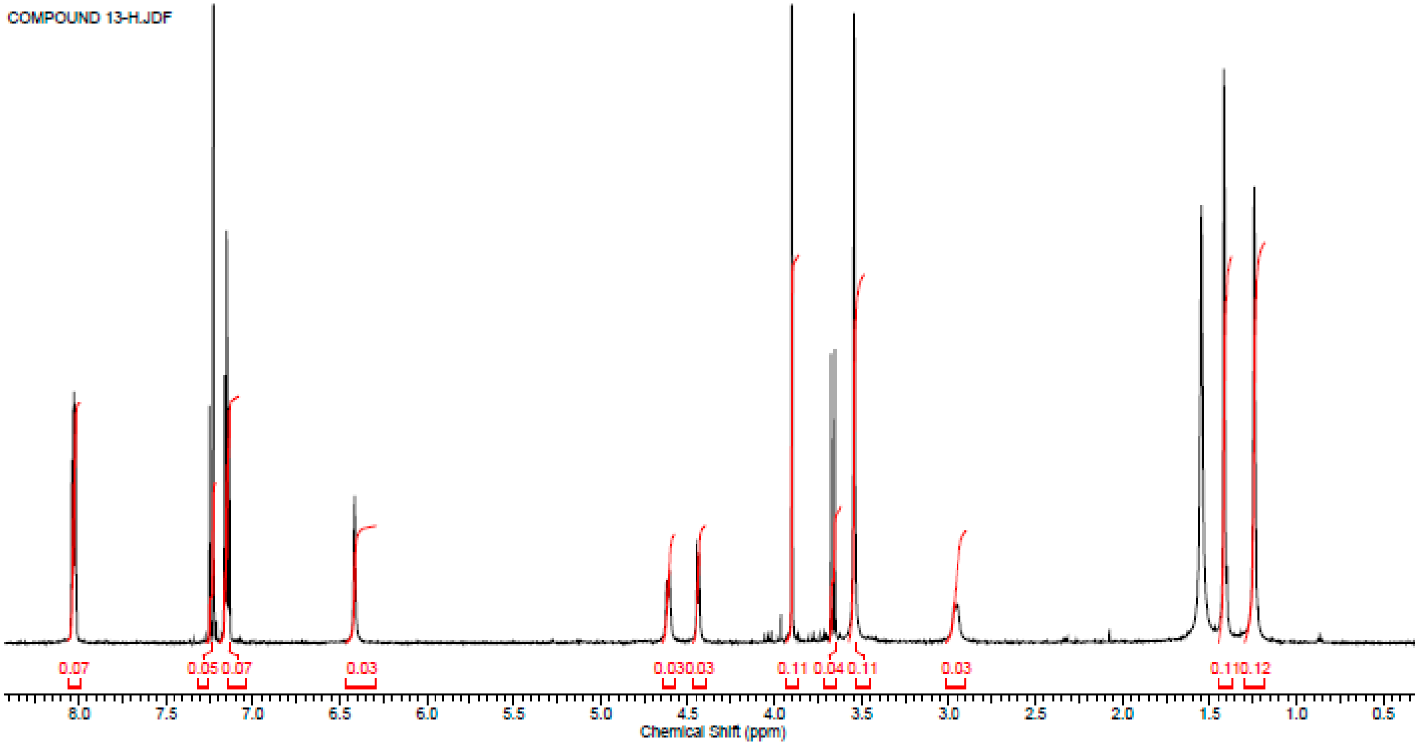

Carbon NMR for compound 13

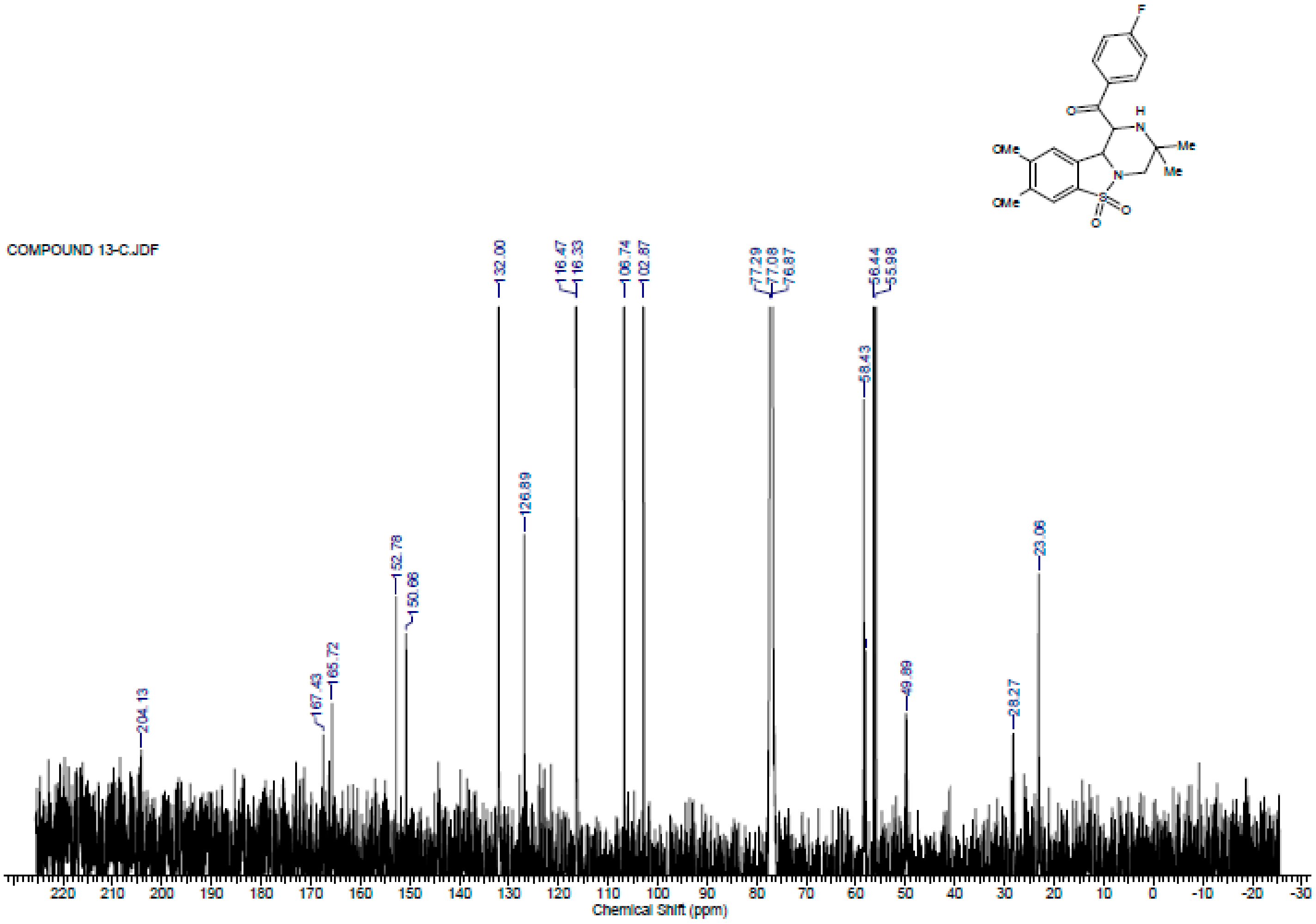

Proton NMR for compound 14

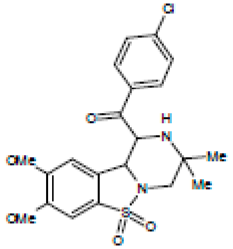

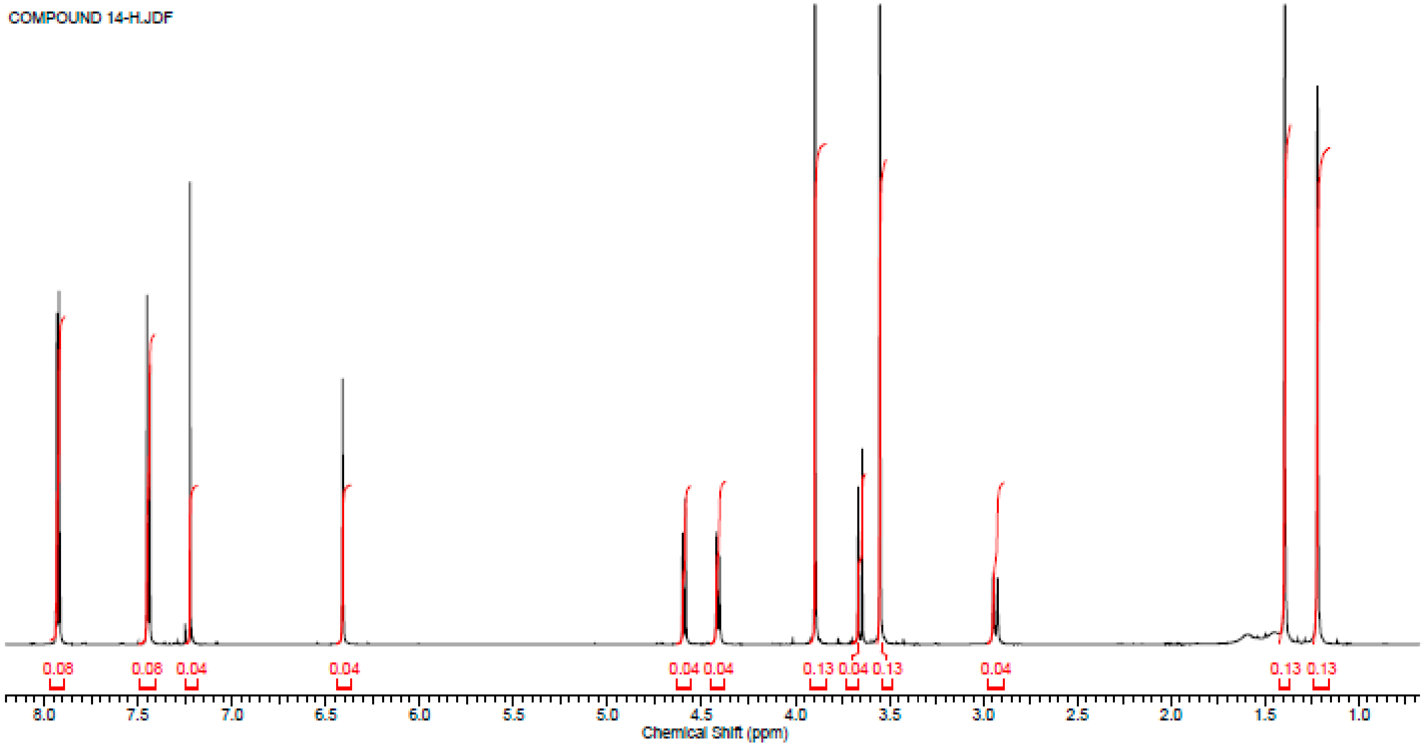

Carbon NMR for compound 14

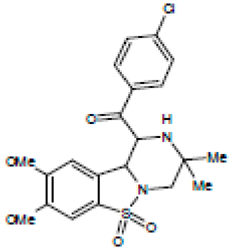

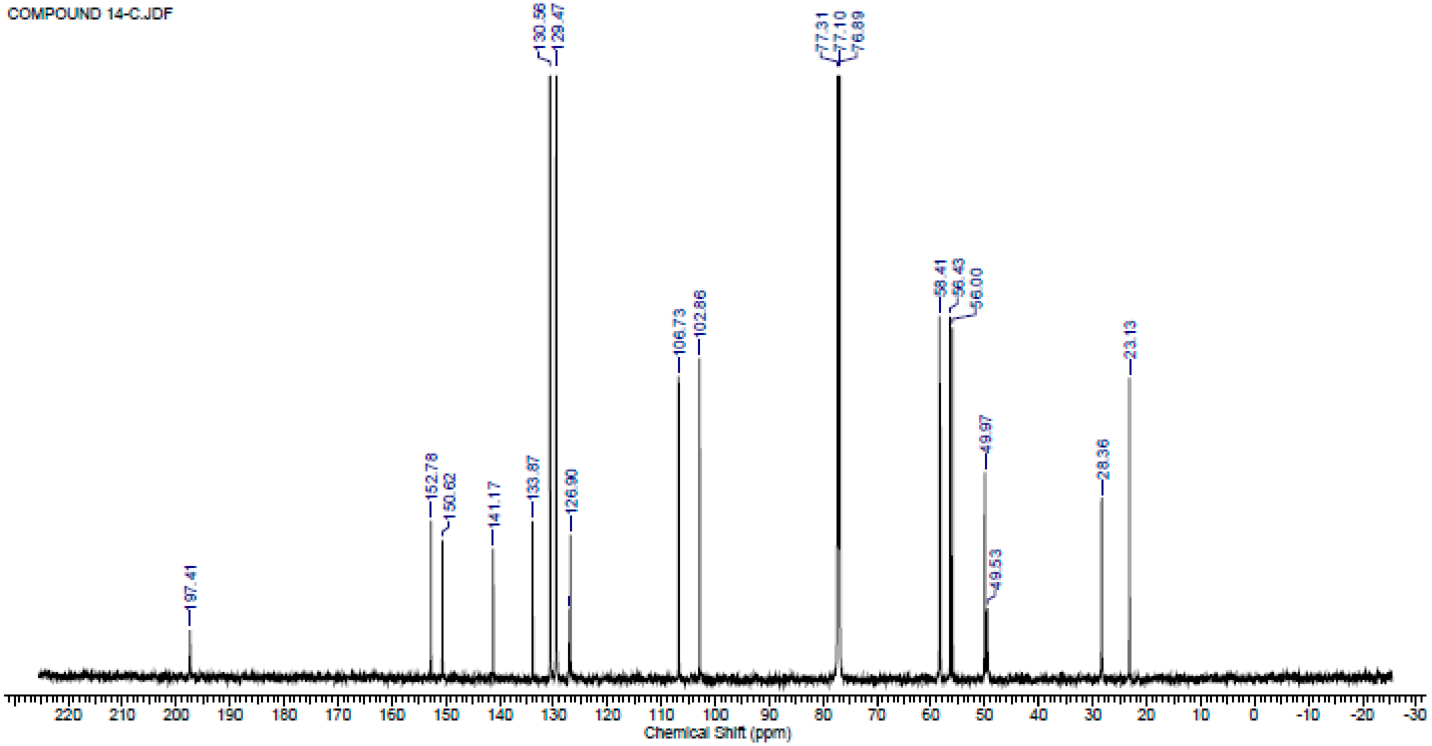

Proton NMR for compound 15

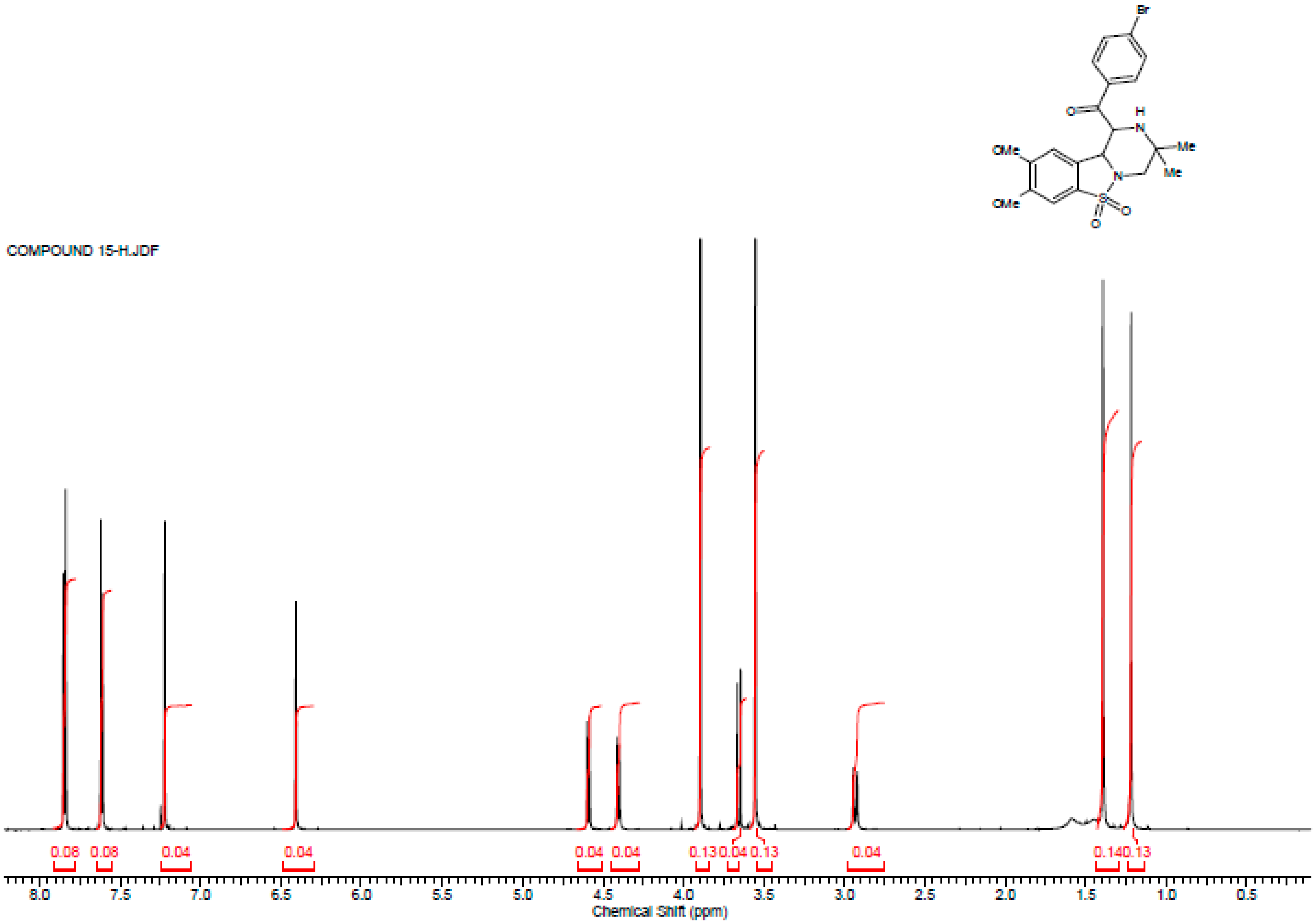

Carbon NMR for compound 15

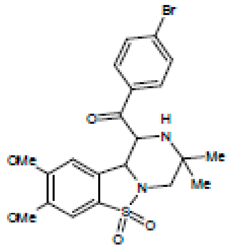

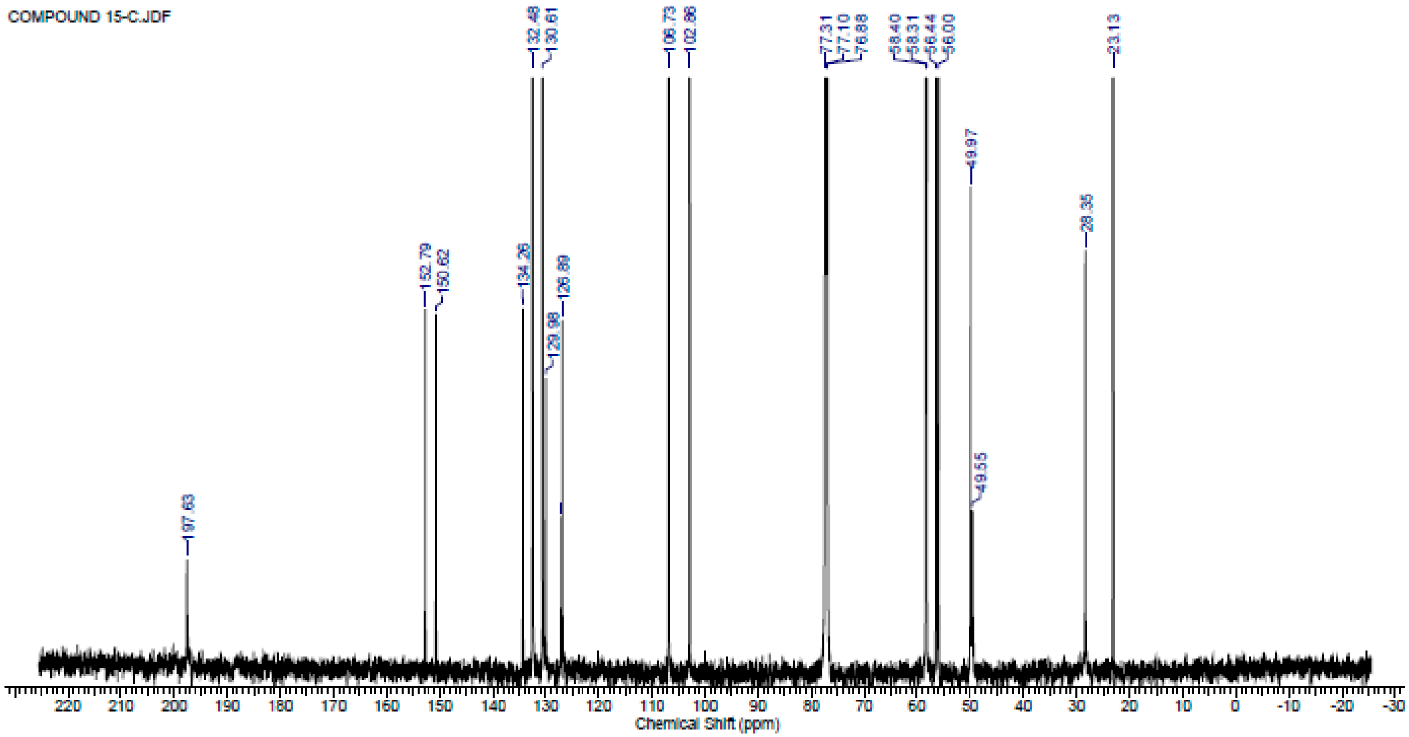

Proton NMR for compound 16

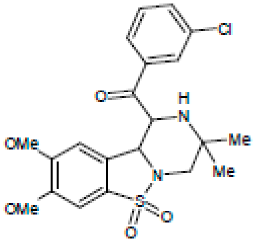

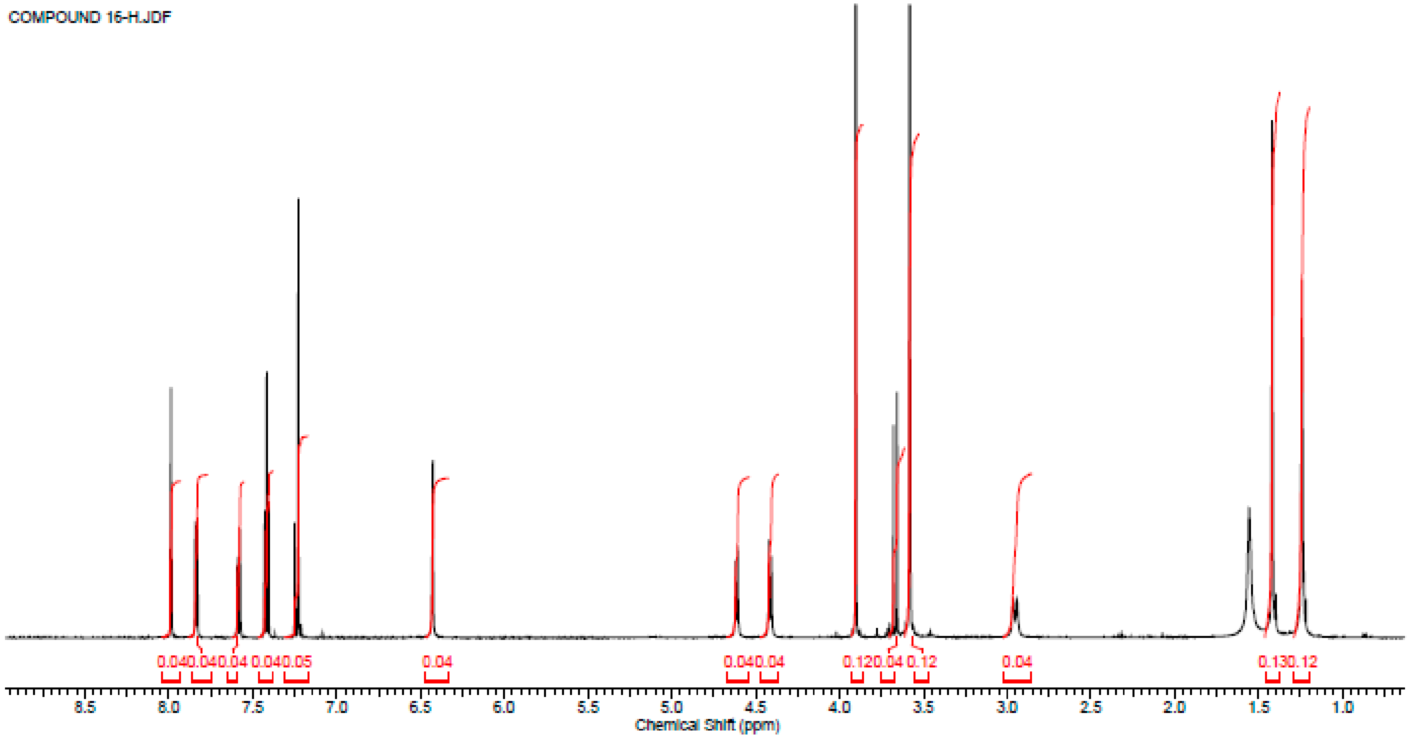

Carbon NMR for compound 16

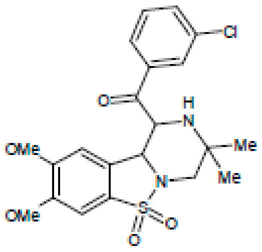

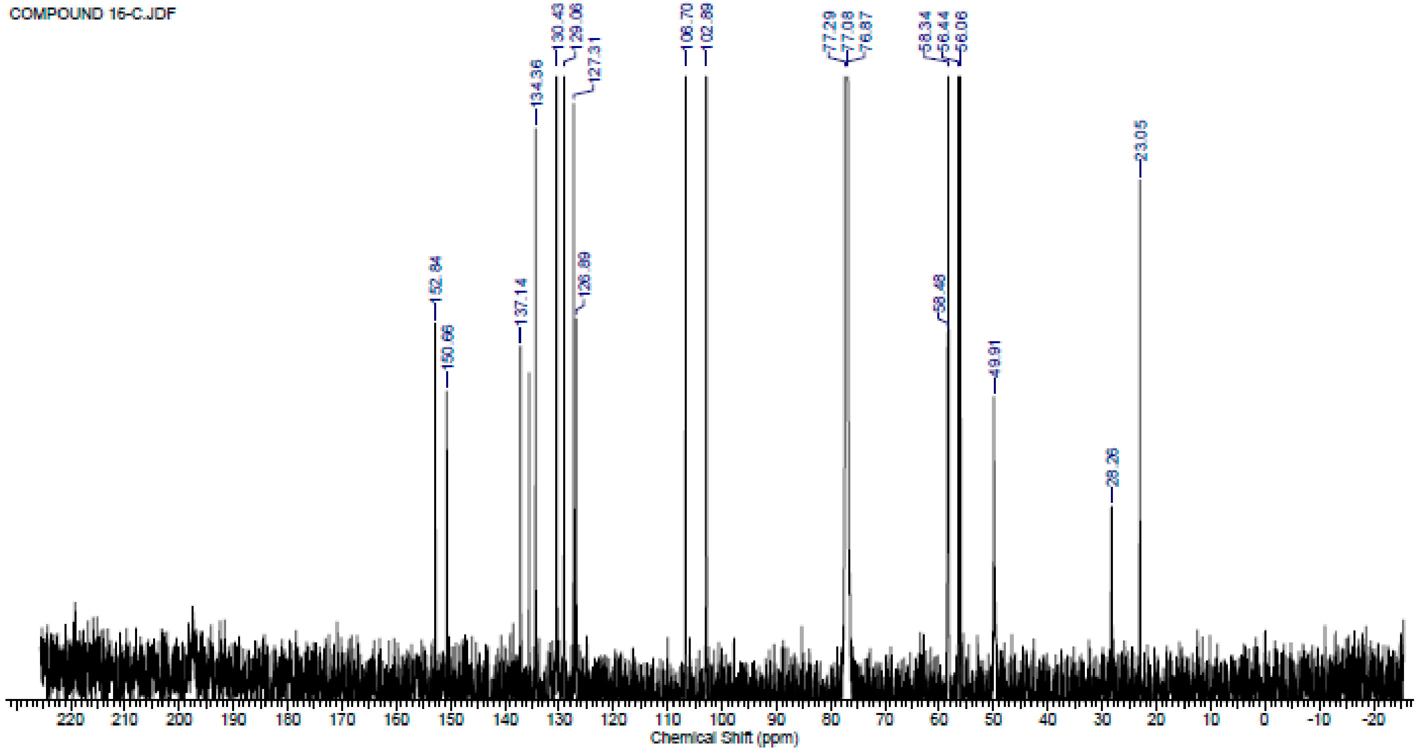

Proton NMR for compound 17

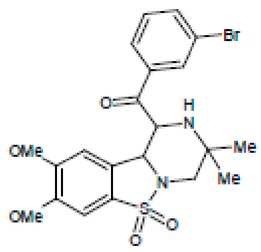

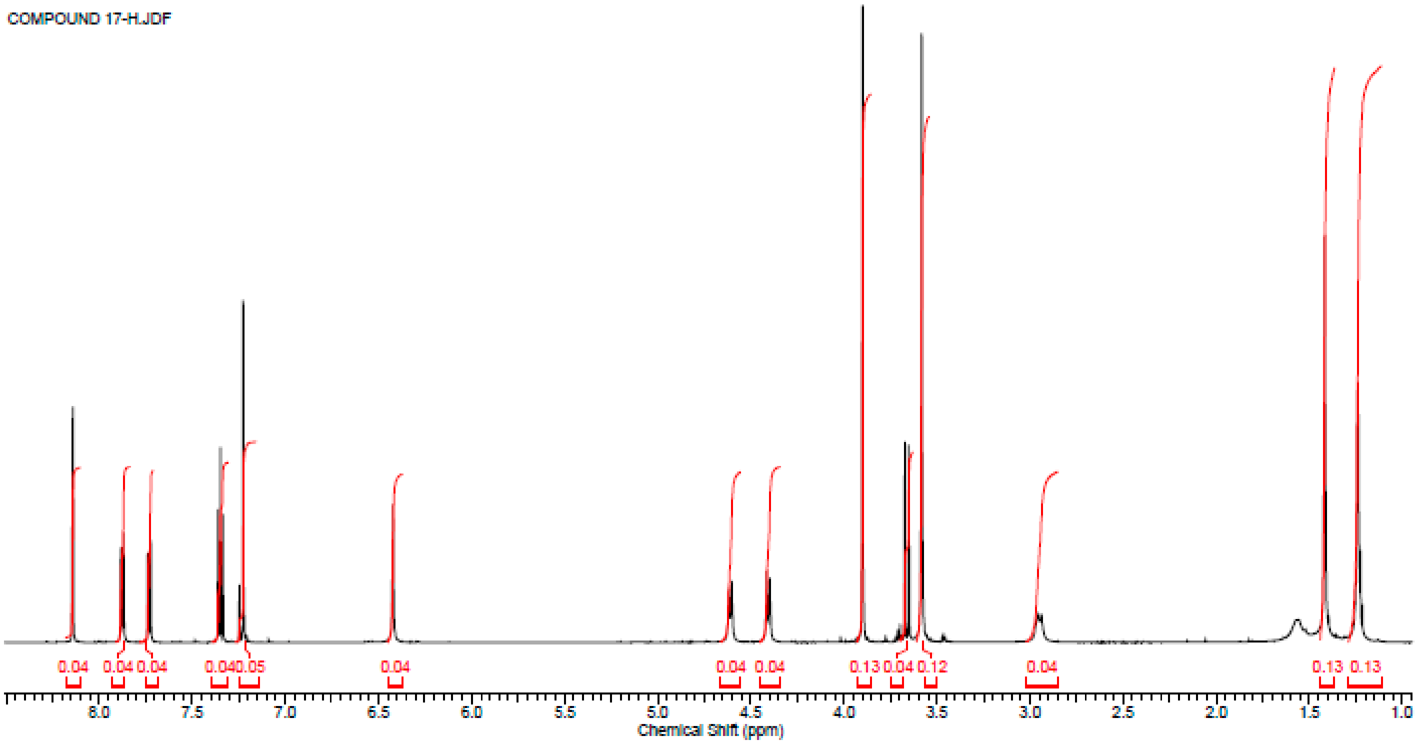

Carbon NMR for compound 17

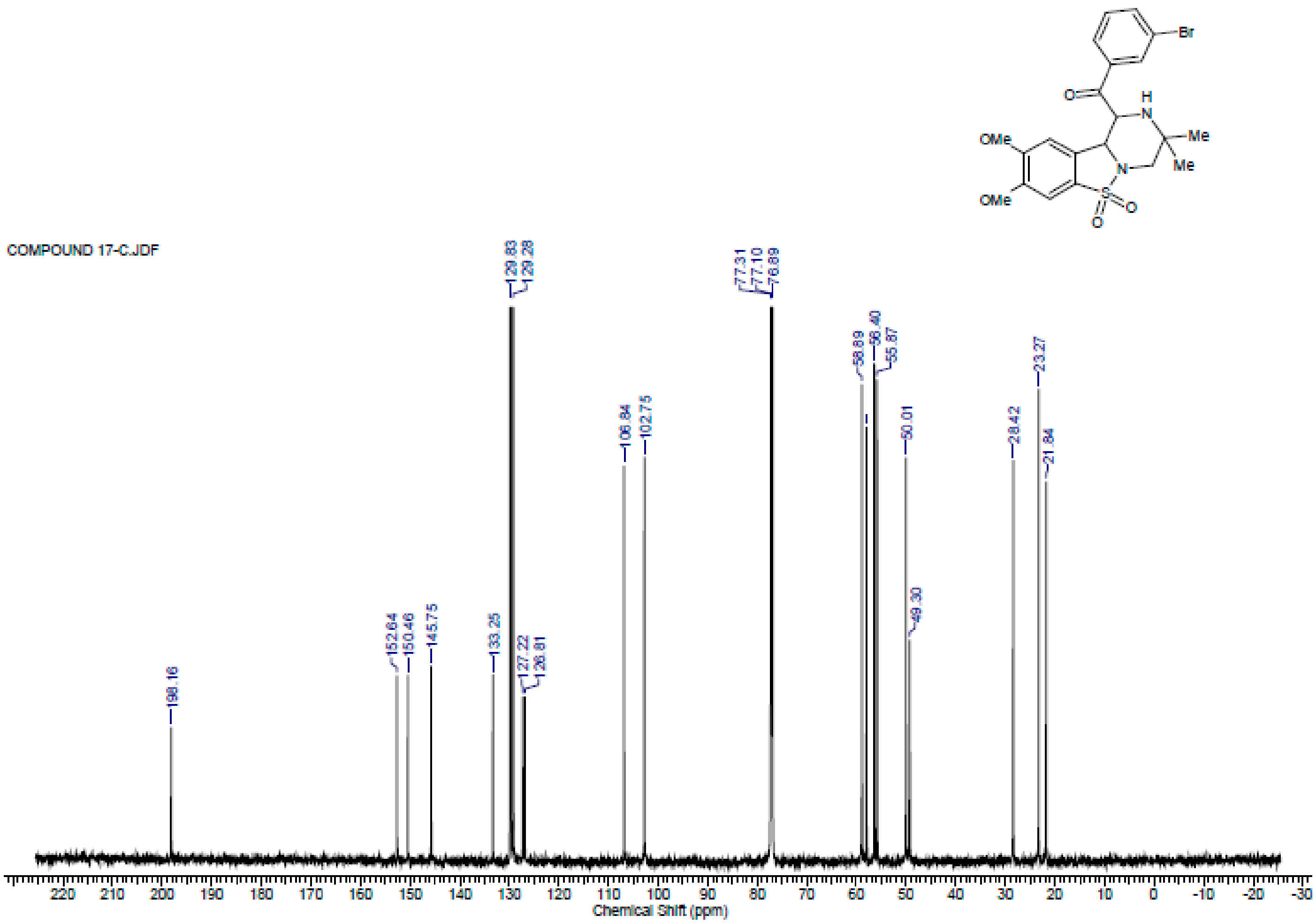

Proton NMR for compound 18

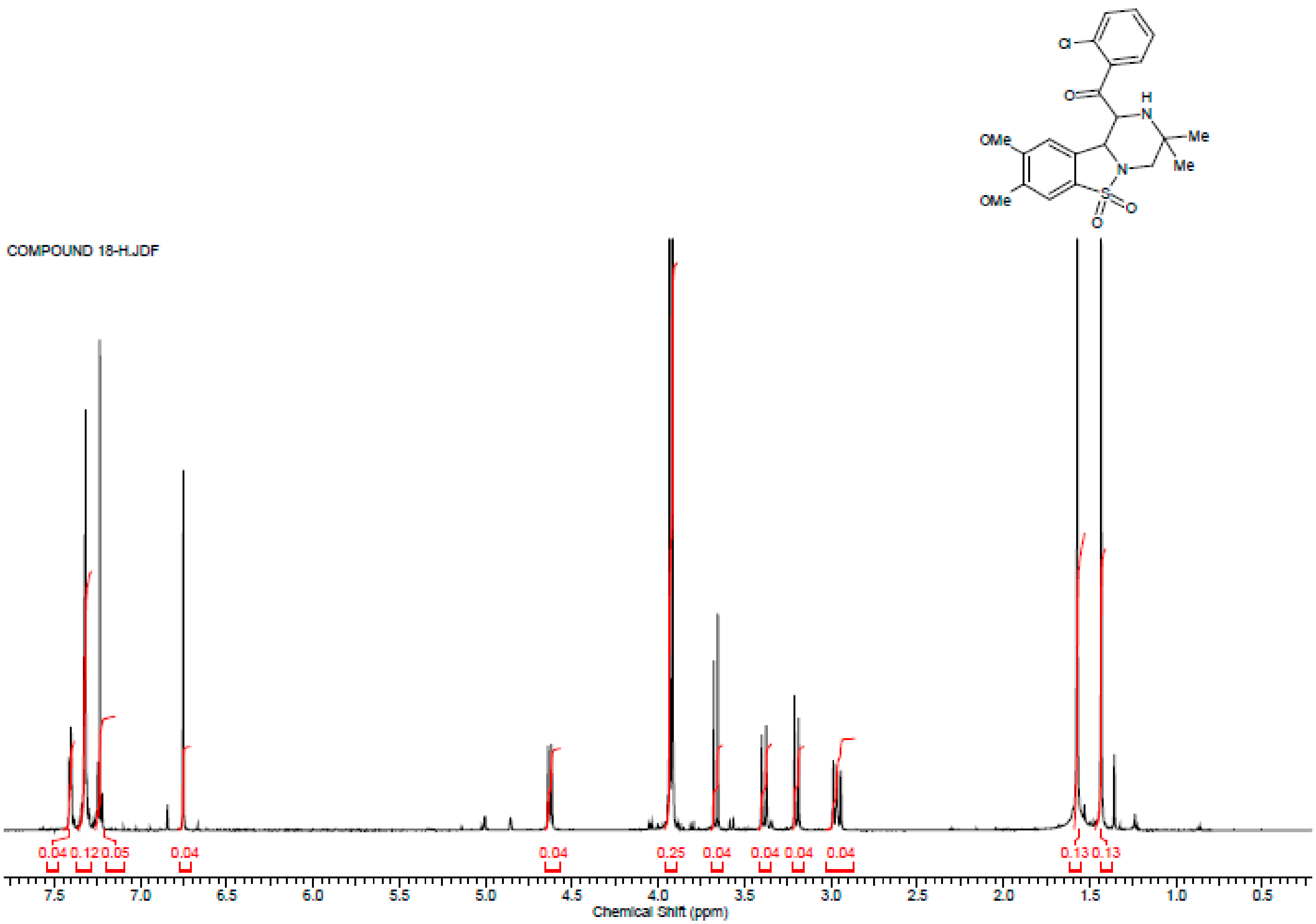

Carbon NMR for compound 18

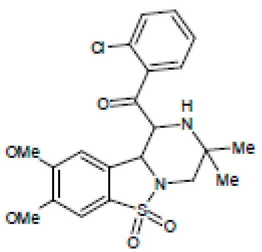

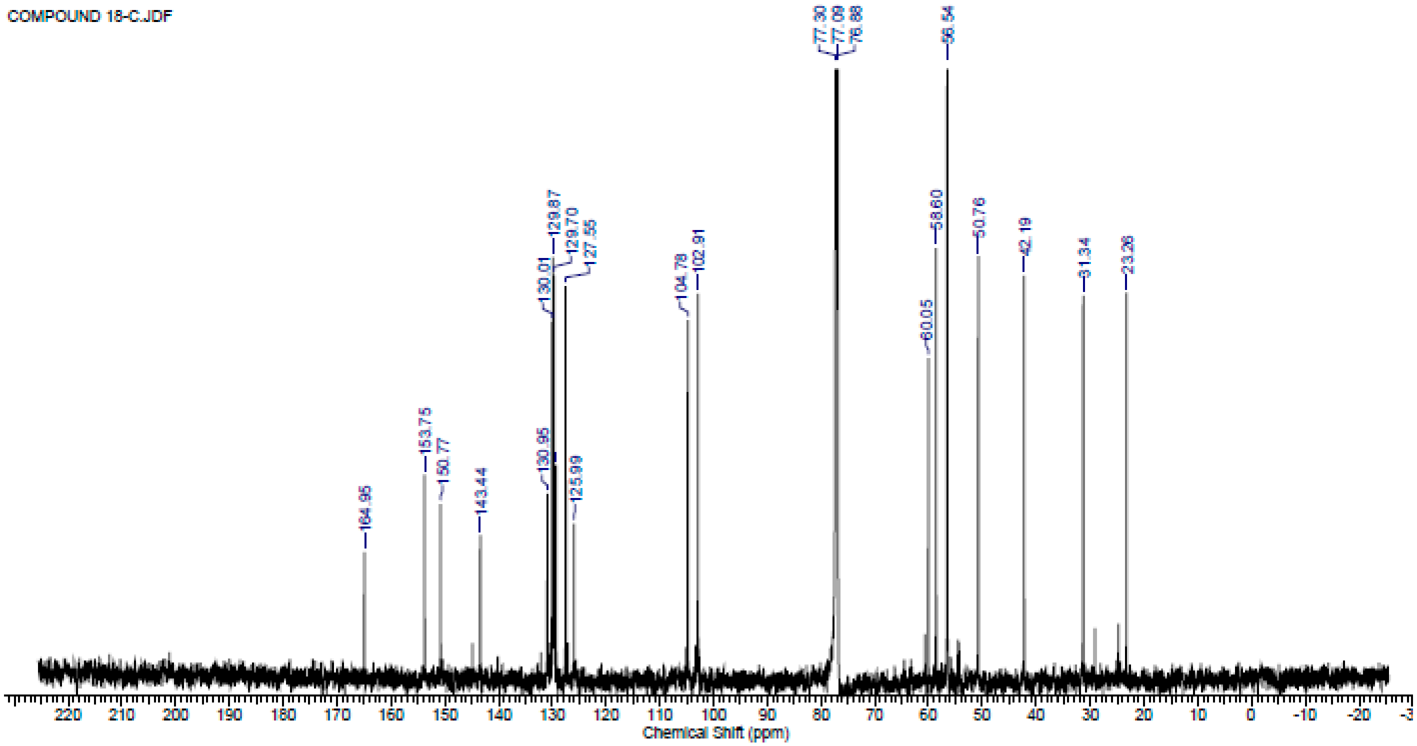

Proton NMR for compound 19

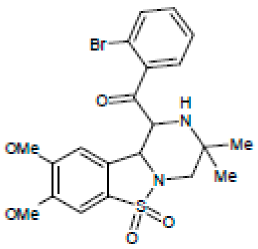

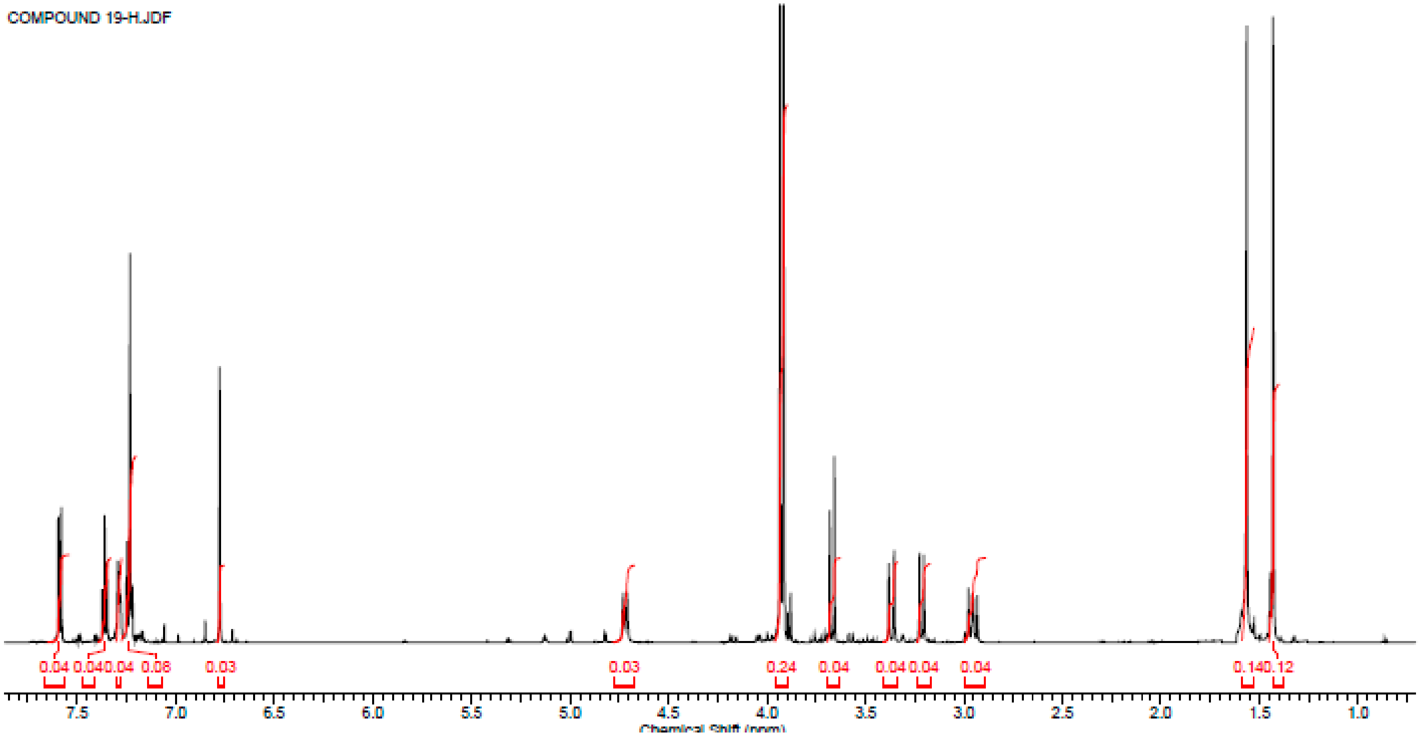

Carbon NMR for compound 19

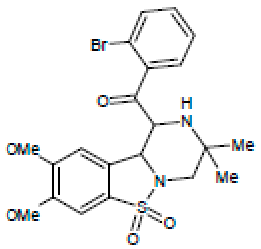

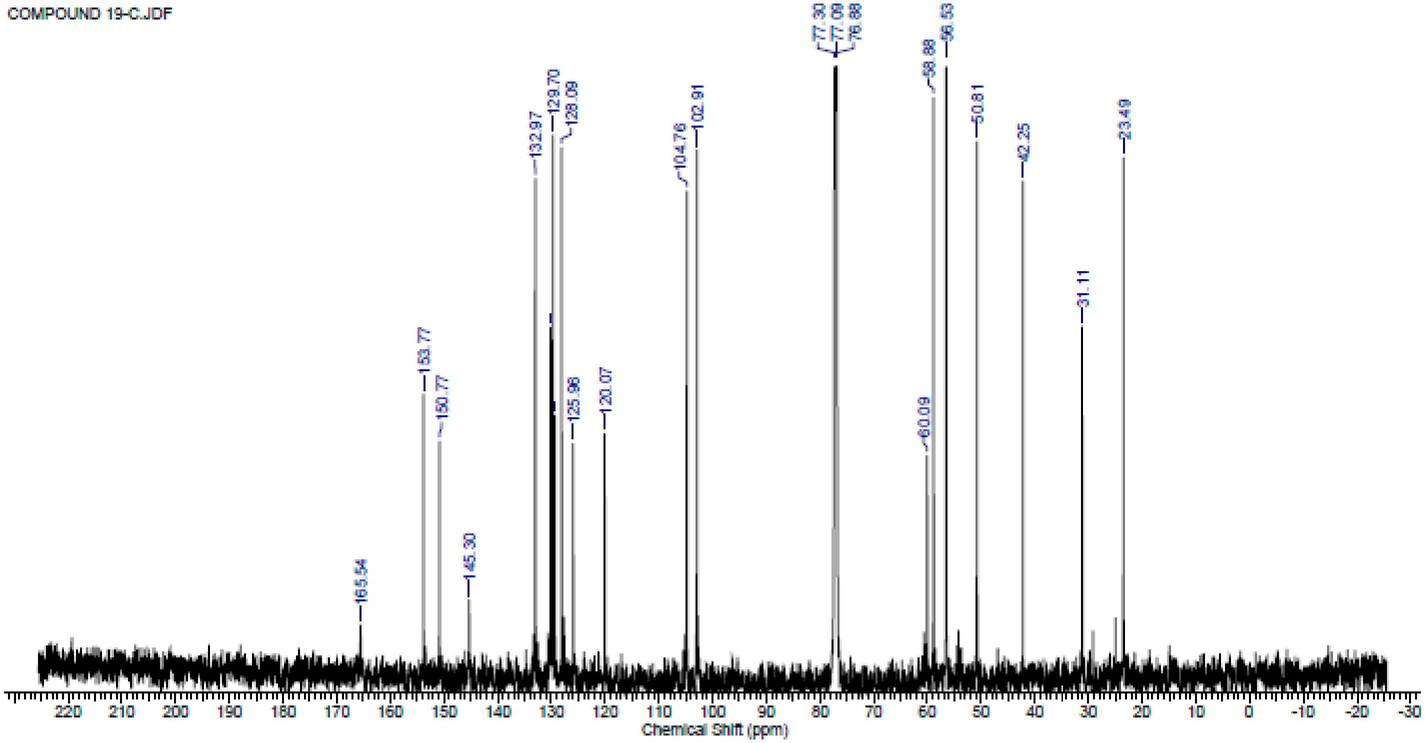

Crystal data and structure refinement for compound **11.**

Identification code	Compound **11**	
Empirical formula	$C_{21}H_{24}N_2O_5S$	
Formula weight	416.5	
Temperature	171(2) K	
Wavelength	0.71073 Å	
Crystal system	Monoclinic	
Space group	P2(1)	
Unit cell dimensions	a = 5.8645(9) Å	α= 90°.
	b = 19.946(3) Å	β= 97.459(3) °.
	c = 8.8732(14) Å	γ = 90°.
Volume	1029.1(3) $Å^3$	
Z	2	
Density (calculated)	1.402 Mg/m^3	
Absorption coefficient	0.199 mm^{-1}	
F(000)	460	
Crystal size	0.30 x 0.19 x 0.08 mm^3	
Theta range for data collection	2.04 to 28.30°.	
Index ranges	-7<=h<=7, -26<=k<=26, -11<=l<=11	
Reflections collected	11737	
Independent reflections	4948 [R(int) = 0.0394]	
Completeness to theta = 28.30°	98.6 %	
Absorption correction	None	
Refinement method	Full-matrix least-squares on F^2	
Data / restraints / parameters	4948 / 3 / 283	
Goodness-of-fit on F^2	0.894	
Final R indices [I>2sigma(I)]	R1 = 0.0419, wR2 = 0.0978	
R indices (all data)	R1 = 0.0523, wR2 = 0.1053	
Absolute structure parameter	0.04(7)	
Largest diff. peak and hole	0.278 and -0.298 e.$Å^{-3}$	

Table i. Atomic coordinates (x 10^4) and equivalent isotropic displacement parameters ($Å^2$x 10^3) for compound **11.**

U(eq) is defined as one third of the trace of the orthogonalized U^{ij} tensor.

	x	y	z	U(eq)
S(1)	260(1)	4525(1)	4207(1)	19(1)
N(1)	-1286(3)	4472(1)	5593(2)	24(1)
N(2)	-3581(4)	4271(1)	8097(2)	22(1)
O(1)	-871(3)	4889(1)	2912(2)	30(1)
O(2)	2508(3)	4773(1)	4770(2)	29(1)
O(3)	2077(3)	2198(1)	1904(2)	29(1)
O(4)	-1094(3)	1656(1)	3240(2)	29(1)
O(5)	-6807(3)	3350(1)	7124(2)	28(1)
C(1)	124(4)	3663(1)	3872(3)	20(1)
C(2)	1398(4)	3307(1)	2911(3)	22(1)
C(3)	958(4)	2624(1)	2747(3)	23(1)
C(4)	-782(4)	2322(1)	3495(3)	22(1)
C(5)	-2039(4)	2700(1)	4406(3)	22(1)
C(6)	-1569(4)	3378(1)	4618(3)	18(1)
C(7)	-2752(4)	3871(1)	5551(3)	20(1)
C(8)	-2006(4)	5066(1)	6367(3)	22(1)
C(9)	-2094(4)	4875(1)	8034(3)	22(1)
C(10)	-2915(4)	3686(1)	7241(3)	20(1)
C(11)	-4816(4)	3161(1)	7311(3)	20(1)
C(12)	-4258(4)	2439(1)	7605(3)	21(1)
C(13)	-2147(5)	2215(1)	8322(3)	30(1)
C(14)	-1770(6)	1538(2)	8607(4)	39(1)
C(15)	-3484(5)	1077(1)	8153(3)	37(1)
C(16)	-5602(5)	1293(1)	7438(3)	32(1)
C(17)	-5998(5)	1963(1)	7170(3)	26(1)
C(18)	3689(5)	2486(1)	1012(3)	30(1)
C(19)	-2774(5)	1339(1)	4041(3)	32(1)
C(20)	297(4)	4725(1)	8852(3)	31(1)
C(21)	-3180(5)	5447(1)	8839(3)	29(1)
O(1W)	-4746(4)	4118(1)	1223(3)	51(1)

Table ii.Bond lengths [Å] and angles [°] for **11.**

S(1)-O(2)	1.4353(18)
S(1)-O(1)	1.4449(18)
S(1)-N(1)	1.6232(19)
S(1)-C(1)	1.747(2)
N(1)-C(8)	1.460(3)
N(1)-C(7)	1.473(3)
N(2)-C(10)	1.472(3)
N(2)-C(9)	1.492(3)
N(2)-H(1N2)	0.75(3)
O(3)-C(3)	1.357(3)
O(3)-C(18)	1.430(3)
O(4)-C(4)	1.356(3)
O(4)-C(19)	1.434(3)
O(5)-C(11)	1.217(3)
C(1)-C(6)	1.385(3)
C(1)-C(2)	1.398(3)
C(2)-C(3)	1.391(3)
C(2)-H(2)	0.9500
C(3)-C(4)	1.421(3)
C(4)-C(5)	1.386(3)
C(5)-C(6)	1.388(3)
C(5)-H(5)	0.9500
C(6)-C(7)	1.511(3)
C(7)-C(10)	1.559(3)
C(7)-H(7)	1.0000
C(8)-C(9)	1.536(3)
C(8)-H(8A)	0.9900
C(8)-H(8B)	0.9900
C(9)-C(20)	1.523(3)
C(9)-C(21)	1.528(3)
C(10)-C(11)	1.536(3)
C(10)-H(10)	1.0000
C(11)-C(12)	1.493(3)
C(12)-C(13)	1.390(4)
C(12)-C(17)	1.410(3)
C(13)-C(14)	1.386(4)
C(13)-H(13)	0.9500
C(14)-C(15)	1.384(4)
C(14)-H(14)	0.9500
C(15)-C(16)	1.388(4)
C(15)-H(15)	0.9500
C(16)-C(17)	1.372(4)
C(16)-H(16)	0.9500
C(17)-H(17)	0.9500
C(18)-H(18A)	0.9800
C(18)-H(18B)	0.9800
C(18)-H(18C)	0.9800
C(19)-H(19A)	0.9800
C(19)-H(19B)	0.9800
C(19)-H(19C)	0.9800
C(20)-H(20A)	0.9800
C(20)-H(20B)	0.9800
C(20)-H(20C)	0.9800

C(21)-H(21A)	0.9800
C(21)-H(21B)	0.9800
C(21)-H(21C)	0.9800
O(1W)-H(1W)	0.83(3)
O(1W)-H(2W)	0.88(3)
O(2)-S(1)-O(1)	114.24(11)
O(2)-S(1)-N(1)	109.70(11)
O(1)-S(1)-N(1)	113.03(11)
O(2)-S(1)-C(1)	114.69(11)
O(1)-S(1)-C(1)	110.66(11)
N(1)-S(1)-C(1)	92.67(11)
C(8)-N(1)-C(7)	118.01(18)
C(8)-N(1)-S(1)	121.80(16)
C(7)-N(1)-S(1)	114.58(15)
C(10)-N(2)-C(9)	115.20(18)
C(10)-N(2)-H(1N2)	110(2)
C(9)-N(2)-H(1N2)	106(2)
C(3)-O(3)-C(18)	117.08(19)
C(4)-O(4)-C(19)	115.83(19)
C(6)-C(1)-C(2)	123.9(2)
C(6)-C(1)-S(1)	110.02(17)
C(2)-C(1)-S(1)	125.98(18)
C(3)-C(2)-C(1)	117.0(2)
C(3)-C(2)-H(2)	121.5
C(1)-C(2)-H(2)	121.5
O(3)-C(3)-C(2)	125.1(2)
O(3)-C(3)-C(4)	114.8(2)
C(2)-C(3)-C(4)	120.1(2)
O(4)-C(4)-C(5)	124.1(2)
O(4)-C(4)-C(3)	115.3(2)
C(5)-C(4)-C(3)	120.6(2)
C(4)-C(5)-C(6)	119.9(2)
C(4)-C(5)-H(5)	120.1
C(6)-C(5)-H(5)	120.1
C(1)-C(6)-C(5)	118.5(2)
C(1)-C(6)-C(7)	113.9(2)
C(5)-C(6)-C(7)	127.6(2)
N(1)-C(7)-C(6)	103.60(18)
N(1)-C(7)-C(10)	106.06(18)
C(6)-C(7)-C(10)	117.59(19)
N(1)-C(7)-H(7)	109.7
C(6)-C(7)-H(7)	109.7
C(10)-C(7)-H(7)	109.7
N(1)-C(8)-C(9)	107.41(18)
N(1)-C(8)-H(8A)	110.2
C(9)-C(8)-H(8A)	110.2
N(1)-C(8)-H(8B)	110.2
C(9)-C(8)-H(8B)	110.2
H(8A)-C(8)-H(8B)	108.5
N(2)-C(9)-C(20)	109.3(2)
N(2)-C(9)-C(21)	107.77(19)
C(20)-C(9)-C(21)	109.8(2)
N(2)-C(9)-C(8)	109.22(19)
C(20)-C(9)-C(8)	111.3(2)
C(21)-C(9)-C(8)	109.4(2)

N(2)-C(10)-C(11)	106.05(18)
N(2)-C(10)-C(7)	111.24(18)
C(11)-C(10)-C(7)	109.62(19)
N(2)-C(10)-H(10)	110.0
C(11)-C(10)-H(10)	110.0
C(7)-C(10)-H(10)	110.0
O(5)-C(11)-C(12)	120.4(2)
O(5)-C(11)-C(10)	118.2(2)
C(12)-C(11)-C(10)	121.4(2)
C(13)-C(12)-C(17)	118.6(2)
C(13)-C(12)-C(11)	123.5(2)
C(17)-C(12)-C(11)	117.9(2)
C(14)-C(13)-C(12)	120.6(3)
C(14)-C(13)-H(13)	119.7
C(12)-C(13)-H(13)	119.7
C(15)-C(14)-C(13)	120.1(3)
C(15)-C(14)-H(14)	119.9
C(13)-C(14)-H(14)	119.9
C(14)-C(15)-C(16)	120.0(3)
C(14)-C(15)-H(15)	120.0
C(16)-C(15)-H(15)	120.0
C(17)-C(16)-C(15)	120.2(3)
C(17)-C(16)-H(16)	119.9
C(15)-C(16)-H(16)	119.9
C(16)-C(17)-C(12)	120.6(3)
C(16)-C(17)-H(17)	119.7
C(12)-C(17)-H(17)	119.7
O(3)-C(18)-H(18A)	109.5
O(3)-C(18)-H(18B)	109.5
H(18A)-C(18)-H(18B)	109.5
O(3)-C(18)-H(18C)	109.5
H(18A)-C(18)-H(18C)	109.5
H(18B)-C(18)-H(18C)	109.5
O(4)-C(19)-H(19A)	109.5
O(4)-C(19)-H(19B)	109.5
H(19A)-C(19)-H(19B)	109.5
O(4)-C(19)-H(19C)	109.5
H(19A)-C(19)-H(19C)	109.5
H(19B)-C(19)-H(19C)	109.5
C(9)-C(20)-H(20A)	109.5
C(9)-C(20)-H(20B)	109.5
H(20A)-C(20)-H(20B)	109.5
C(9)-C(20)-H(20C)	109.5
H(20A)-C(20)-H(20C)	109.5
H(20B)-C(20)-H(20C)	109.5
C(9)-C(21)-H(21A)	109.5
C(9)-C(21)-H(21B)	109.5
H(21A)-C(21)-H(21B)	109.5
C(9)-C(21)-H(21C)	109.5
H(21A)-C(21)-H(21C)	109.5
H(21B)-C(21)-H(21C)	109.5
H(1W)-O(1W)-H(2W)	97(4)

Symmetry transformations used to generate equivalent atoms:

Table iii. Anisotropic displacement parameters ($Å^2 x 10^3$) for compound **11.** The anisotropic displacement factor exponent takes the form: -2 ^{2}[$h^2 a^{*2}U^{11}$+ 2 h k a* b* U^{12}]

	U^{11}	U^{22}	U^{33}	U^{23}	U^{13}	U^{12}
S(1)	20(1)	16(1)	22(1)	0(1)	6(1)	0(1)
N(1)	31(1)	18(1)	27(1)	-5(1)	16(1)	-5(1)
N(2)	21(1)	22(1)	24(1)	-3(1)	7(1)	0(1)
O(1)	40(1)	23(1)	26(1)	4(1)	4(1)	3(1)
O(2)	20(1)	23(1)	43(1)	-5(1)	4(1)	-3(1)
O(3)	36(1)	21(1)	35(1)	-5(1)	20(1)	0(1)
O(4)	37(1)	17(1)	36(1)	-4(1)	17(1)	-3(1)
O(5)	18(1)	24(1)	41(1)	2(1)	6(1)	0(1)
C(1)	22(1)	15(1)	23(1)	-2(1)	3(1)	-1(1)
C(2)	24(1)	20(1)	22(1)	-1(1)	8(1)	-2(1)
C(3)	26(1)	22(1)	20(1)	-2(1)	7(1)	2(1)
C(4)	28(1)	17(1)	22(1)	-1(1)	3(1)	0(1)
C(5)	22(1)	20(1)	24(1)	-2(1)	7(1)	-4(1)
C(6)	20(1)	18(1)	17(1)	0(1)	4(1)	2(1)
C(7)	21(1)	17(1)	24(1)	-2(1)	6(1)	-1(1)
C(8)	24(1)	17(1)	26(1)	-5(1)	8(1)	-1(1)
C(9)	21(1)	21(1)	26(1)	-4(1)	9(1)	-2(1)
C(10)	20(1)	19(1)	21(1)	-2(1)	6(1)	-1(1)
C(11)	21(1)	21(1)	20(1)	0(1)	5(1)	-2(1)
C(12)	24(1)	22(1)	20(1)	0(1)	8(1)	-2(1)
C(13)	26(1)	27(1)	35(1)	7(1)	3(1)	-1(1)
C(14)	41(2)	32(2)	45(2)	11(1)	6(1)	8(1)
C(15)	48(2)	22(1)	43(2)	9(1)	18(1)	5(1)
C(16)	40(2)	25(1)	34(1)	-1(1)	13(1)	-7(1)
C(17)	27(1)	25(1)	28(1)	0(1)	8(1)	-3(1)
C(18)	32(1)	33(1)	28(1)	-3(1)	15(1)	0(1)
C(19)	35(2)	19(1)	45(2)	0(1)	16(1)	-4(1)
C(20)	22(1)	35(1)	34(1)	-5(1)	3(1)	-4(1)
C(21)	35(1)	24(1)	31(1)	-7(1)	12(1)	-1(1)
O(1W)	51(1)	72(2)	30(1)	8(1)	6(1)	-29(1)

Table iv. Hydrogen coordinates (x 10^4) and isotropic displacement parameters ($Å^2$x 10^3) for compound **11.**

	x	y	z	U(eq)
H(2)	2514	3522	2392	26
H(5)	-3220	2496	4884	26
H(7)	-4319	3977	5018	24
H(8A)	-3541	5216	5888	26
H(8B)	-898	5436	6302	26
H(10)	-1409	3505	7729	24
H(13)	-954	2528	8620	35
H(14)	-331	1390	9114	47
H(15)	-3211	612	8330	44
H(16)	-6781	976	7133	39
H(17)	-7456	2108	6688	32
H(18A)	4392	2130	466	45
H(18B)	4888	2723	1679	45
H(18C)	2892	2802	279	45
H(19A)	-2869	861	3782	48
H(19B)	-4277	1550	3751	48
H(19C)	-2325	1390	5138	48
H(20A)	187	4606	9912	46
H(20B)	1274	5122	8821	46
H(20C)	969	4350	8349	46
H(21A)	-4708	5547	8300	44
H(21B)	-2207	5847	8845	44
H(21C)	-3319	5315	9887	44
H(1W)	-4250(80)	4180(30)	400(40)	95(17)
H(2W)	-3700(60)	4360(20)	1790(40)	73(13)
H(1N2)	-4770(50)	4381(15)	7780(30)	24(8)

Table v. Torsion angles [°] for compound **11.**

O(2)-S(1)-N(1)-C(8)	67.0(2)
O(1)-S(1)-N(1)-C(8)	-61.7(2)
C(1)-S(1)-N(1)-C(8)	-175.51(19)
O(2)-S(1)-N(1)-C(7)	-139.92(17)
O(1)-S(1)-N(1)-C(7)	91.33(18)
C(1)-S(1)-N(1)-C(7)	-22.48(18)
O(2)-S(1)-C(1)-C(6)	127.07(17)
O(1)-S(1)-C(1)-C(6)	-101.91(18)
N(1)-S(1)-C(1)-C(6)	13.94(18)
O(2)-S(1)-C(1)-C(2)	-57.2(2)
O(1)-S(1)-C(1)-C(2)	73.8(2)
N(1)-S(1)-C(1)-C(2)	-170.3(2)
C(6)-C(1)-C(2)-C(3)	-2.0(4)
S(1)-C(1)-C(2)-C(3)	-177.14(19)
C(18)-O(3)-C(3)-C(2)	-5.8(4)
C(18)-O(3)-C(3)-C(4)	174.1(2)
C(1)-C(2)-C(3)-O(3)	-177.8(2)
C(1)-C(2)-C(3)-C(4)	2.3(3)
C(19)-O(4)-C(4)-C(5)	-2.9(3)
C(19)-O(4)-C(4)-C(3)	177.3(2)
O(3)-C(3)-C(4)-O(4)	-0.9(3)

C(2)-C(3)-C(4)-O(4)	179.0(2)
O(3)-C(3)-C(4)-C(5)	179.3(2)
C(2)-C(3)-C(4)-C(5)	-0.7(4)
O(4)-C(4)-C(5)-C(6)	179.0(2)
C(3)-C(4)-C(5)-C(6)	-1.3(4)
C(2)-C(1)-C(6)-C(5)	0.0(4)
S(1)-C(1)-C(6)-C(5)	175.83(19)
C(2)-C(1)-C(6)-C(7)	-178.1(2)
S(1)-C(1)-C(6)-C(7)	-2.3(2)
C(4)-C(5)-C(6)-C(1)	1.7(3)
C(4)-C(5)-C(6)-C(7)	179.5(2)
C(8)-N(1)-C(7)-C(6)	177.43(18)
S(1)-N(1)-C(7)-C(6)	23.3(2)
C(8)-N(1)-C(7)-C(10)	-58.1(3)
S(1)-N(1)-C(7)-C(10)	147.74(15)
C(1)-C(6)-C(7)-N(1)	-12.1(3)
C(5)-C(6)-C(7)-N(1)	170.0(2)
C(1)-C(6)-C(7)-C(10)	-128.7(2)
C(5)-C(6)-C(7)-C(10)	53.4(3)
C(7)-N(1)-C(8)-C(9)	60.8(3)
S(1)-N(1)-C(8)-C(9)	-147.06(17)
C(10)-N(2)-C(9)-C(20)	-66.5(3)
C(10)-N(2)-C(9)-C(21)	174.2(2)
C(10)-N(2)-C(9)-C(8)	55.5(3)
N(1)-C(8)-C(9)-N(2)	-53.9(2)
N(1)-C(8)-C(9)-C(20)	66.9(2)
N(1)-C(8)-C(9)-C(21)	-171.64(19)
C(9)-N(2)-C(10)-C(11)	-173.64(19)
C(9)-N(2)-C(10)-C(7)	-54.5(3)
N(1)-C(7)-C(10)-N(2)	50.8(2)
C(6)-C(7)-C(10)-N(2)	166.10(19)
N(1)-C(7)-C(10)-C(11)	167.82(18)
C(6)-C(7)-C(10)-C(11)	
